# Epidemiology and Reporting Characteristics of Systematic Reviews of Biomedical Research: A Cross-Sectional Study

**DOI:** 10.1371/journal.pmed.1002028

**Published:** 2016-05-24

**Authors:** Matthew J. Page, Larissa Shamseer, Douglas G. Altman, Jennifer Tetzlaff, Margaret Sampson, Andrea C. Tricco, Ferrán Catalá-López, Lun Li, Emma K. Reid, Rafael Sarkis-Onofre, David Moher

**Affiliations:** 1 School of Public Health and Preventive Medicine, Monash University, Melbourne, Victoria, Australia; 2 School of Social and Community Medicine, University of Bristol, Bristol, United Kingdom; 3 Clinical Epidemiology Program, Ottawa Hospital Research Institute, Ottawa, Ontario, Canada; 4 School of Epidemiology, Public Health and Preventive Medicine, Faculty of Medicine, University of Ottawa, Ottawa, Ontario, Canada; 5 Centre for Statistics in Medicine, University of Oxford, Oxford, United Kingdom; 6 Children’s Hospital of Eastern Ontario, Ottawa, Ontario, Canada; 7 Li Ka Shing Knowledge Institute of St. Michael’s Hospital, Toronto, Ontario, Canada; 8 Epidemiology Division, Dalla Lana School of Public Health, University of Toronto, Toronto, Ontario, Canada; 9 Department of Medicine, University of Valencia/INCLIVA Health Research Institute and Centro de Investigación en Red de Salud Mental, Valencia, Spain; 10 First Clinical College, Lanzhou University, Lanzhou, China; 11 Department of Pharmacy, Vancouver General Hospital, Vancouver, British Columbia, Canada; 12 Graduate Program in Dentistry, Federal University of Pelotas, Pelotas, Brazil; University of Bern, SWITZERLAND

## Abstract

**Background:**

Systematic reviews (SRs) can help decision makers interpret the deluge of published biomedical literature. However, a SR may be of limited use if the methods used to conduct the SR are flawed, and reporting of the SR is incomplete. To our knowledge, since 2004 there has been no cross-sectional study of the prevalence, focus, and completeness of reporting of SRs across different specialties. Therefore, the aim of our study was to investigate the epidemiological and reporting characteristics of a more recent cross-section of SRs.

**Methods and Findings:**

We searched MEDLINE to identify potentially eligible SRs indexed during the month of February 2014. Citations were screened using prespecified eligibility criteria. Epidemiological and reporting characteristics of a random sample of 300 SRs were extracted by one reviewer, with a 10% sample extracted in duplicate. We compared characteristics of Cochrane versus non-Cochrane reviews, and the 2014 sample of SRs versus a 2004 sample of SRs. We identified 682 SRs, suggesting that more than 8,000 SRs are being indexed in MEDLINE annually, corresponding to a 3-fold increase over the last decade. The majority of SRs addressed a therapeutic question and were conducted by authors based in China, the UK, or the US; they included a median of 15 studies involving 2,072 participants. Meta-analysis was performed in 63% of SRs, mostly using standard pairwise methods. Study risk of bias/quality assessment was performed in 70% of SRs but was rarely incorporated into the analysis (16%). Few SRs (7%) searched sources of unpublished data, and the risk of publication bias was considered in less than half of SRs. Reporting quality was highly variable; at least a third of SRs did not report use of a SR protocol, eligibility criteria relating to publication status, years of coverage of the search, a full Boolean search logic for at least one database, methods for data extraction, methods for study risk of bias assessment, a primary outcome, an abstract conclusion that incorporated study limitations, or the funding source of the SR. Cochrane SRs, which accounted for 15% of the sample, had more complete reporting than all other types of SRs. Reporting has generally improved since 2004, but remains suboptimal for many characteristics.

**Conclusions:**

An increasing number of SRs are being published, and many are poorly conducted and reported. Strategies are needed to help reduce this avoidable waste in research.

## Abstract 2

In a cross-sectional manuscript analysis, David Moher and colleagues score the prevalence, quality of conduct and completeness of reporting among systematic reviews published across medical disciplines in 2014.

## Author Summary

**Why Was This Study Done?:**

- Decisions in health care, such as which treatment to recommend or which test to order, should be based on evidence from all available research studies, rather than the results of the largest or most recent study.
- Systematic reviews, which explicitly use methods to identify, select, critically appraise, and synthesize the results of all existing studies of a given question, are considered the highest level of evidence for decision makers.
- We wanted to know how many systematic reviews of biomedical research are being published, what questions they are addressing, and how well the methods are reported, since information of this sort has not been collected since 2004.

**What Did the Researchers Do and Find?:**

- We looked for all systematic reviews added to the main bibliographic database for biomedical literature during one month (February 2014), and recorded the characteristics of these reviews.
- We found 682 systematic reviews—a 3-fold increase over the last decade—that addressed a wide range of topics.
- In many cases, important aspects of the methods used were not reported (for example, at least a third of the reviews did not report how they searched for studies or how they assessed the quality of the included studies), unpublished data was rarely sought, and at least a third of the reviews used statistical methods discouraged by leading organizations that have developed guidance for systematic reviews (for example, Cochrane and the Institute of Medicine).

**What Do These Findings Mean?:**

- We conclude that systematic reviews have become increasingly popular and that, similar to a decade ago, the quality of conduct and reporting varies widely; therefore, readers should not accept the findings of systematic reviews uncritically.
- We recommend a number of strategies to improve the value of systematic reviews, such as the development of software to facilitate better reporting, certified training for journal editors in how to implement the use of reporting guidelines such as PRISMA (http://prisma-statement.org/), and formal training of biomedical researchers in research design and analysis.

## Introduction

Biomedical and public health research is conducted on a massive scale; for instance, more than 750,000 publications were indexed in MEDLINE in 2014 [1]. Systematic reviews (SRs) of research studies can help users make sense of this vast literature, by synthesizing the results of studies that address a particular question, in a rigorous and replicable way. By examining the accumulated body of evidence rather than the results of single studies, SRs can provide more reliable results for a range of health care enquiries (e.g., what are the benefits and harms of therapeutic interventions, what is the accuracy of diagnostic tests) [2,3]. SRs can also identify gaps in knowledge and inform future research agendas. However, a SR may be of limited use to decision makers if the methods used to conduct the SR are flawed, and reporting of the SR is incomplete [4,5].

Moher et al. previously investigated the prevalence of SRs in the biomedical literature and their quality of reporting [6]. A search of MEDLINE in November 2004 identified 300 SRs indexed in that month, which corresponded to an annual publication prevalence of 2,500 SRs. The majority of SRs (71%) focused on a therapeutic question (as opposed to a diagnosis, prognosis, or epidemiological question), and 20% were Cochrane SRs. The reporting quality varied, with only 66% reporting the years of their search, 69% assessing study risk of bias/quality, 50% using the term “systematic review” or “meta-analysis” in the title or abstract, 23% formally assessing evidence for publication bias, and 60% reporting the funding source of the SR.

The publication landscape for SRs has changed considerably in the subsequent decade. Major events include the publication of the PRISMA reporting guidelines for SRs [7,8] and SR abstracts [9] and their subsequent endorsement in top journals, the launch of the Institute of Medicine’s standards for SRs of comparative effectiveness research [10], methodological developments such as a new tool to assess the risk of bias in randomized trials included in SRs [11], and the proliferation of open-access journals to disseminate health and medical research findings, in particular *Systematic Reviews*, a journal specifically for completed SRs, their protocols, and associated research [12]. Other studies have examined in more recent samples either the prevalence of SRs (e.g., [13]) or reporting characteristics of SRs in specific fields (e.g., physical therapy [14], complementary and alternative medicine [15], and radiology [16]). However, to our knowledge, since the 2004 sample, there has been no cross-sectional study of the characteristics of SRs across different specialties. Therefore, we considered it timely to explore the prevalence and focus of a more recent cross-section of SRs, and to assess whether reporting quality has improved over time.

The primary objective of this study was to investigate the epidemiological and reporting characteristics of SRs indexed in MEDLINE during the month of February 2014. Secondary objectives were to explore (1) how the characteristics of different types of reviews (e.g., therapeutic, epidemiology, diagnosis) vary; (2) whether the reporting quality of therapeutic SRs is associated with whether a SR was a Cochrane review and with self-reported use of the PRISMA Statement; and (3) how the current sample of SRs differs to the sample of SRs in 2004.

## Methods

### Study Protocol

We prespecified our methods in a study protocol (S1 Protocol).

### Eligibility Criteria

We included articles that we considered to meet the PRISMA-P definition of a SR [17,18], that is, articles that explicitly stated methods to identify studies (i.e., a search strategy), explicitly stated methods of study selection (e.g., eligibility criteria and selection process), and explicitly described methods of synthesis (or other type of summary). We did not exclude SRs based on the type of methods they used (e.g., an assessment of the validity of findings of included studies could be reported using a structured tool or informally in the limitations section of the Discussion). Also, we did not exclude SRs based on the level of detail they reported about their methods (e.g., authors could present a line-by-line Boolean search strategy or just list the key words they used in the search). Further, we included articles regardless of the SR question (e.g., therapeutic, diagnostic, etiology) and the types of studies included (e.g., quantitative or qualitative). We included only published SRs that were written in English, to be consistent with the previous study [6].

We used the PRISMA-P definition of SRs because it is in accordance with the definition reported in the PRISMA Statement [7] and with that used by Cochrane [19], by the Agency for Healthcare Research and Quality’s Evidence-based Practice Centers Program [20], and in the 2011 guidance from the Institute of Medicine [10]. Further, the SR definition used by Moher et al. for the 2004 sample (“the authors’ stated objective was to summarize evidence from multiple studies and the article described explicit methods, regardless of the details provided”) [6] ignores the evolution of SR terminology over time.

We excluded the following types of articles: narrative/non-systematic literature reviews; non-systematic literature reviews with meta-analysis or meta-synthesis, where the authors conducted a meta-analysis or meta-synthesis of studies but did not use SR methods to identify and select the studies; articles described by the authors as “rapid reviews” or literature reviews produced using accelerated or abbreviated SR methods; overviews of reviews (or umbrella reviews); scoping reviews; methodology studies that included a systematic search for studies to evaluate some aspect of conduct/reporting (e.g., assessments of the extent to which all trials published in 2012 adhered to the CONSORT Statement); and protocols or summaries of SRs.

### Searching

We searched for SRs indexed throughout one calendar month. We selected February 2014, as it was the month closest to when the protocol for this study was drafted. We searched Ovid MEDLINE In-Process & Other Non-Indexed Citations and Ovid MEDLINE 1946 to Present using the search strategy reported by Moher et al. [6]: (1) 201402$.ed; (2) limit 1 to English; (3) 2 and (cochrane database of systematic reviews.jn. or search.tw. or metaanalysis.pt. or medline.tw. or systematic review.tw. or ((metaanalysis.mp,pt. or review.pt. or search$.tw.) and methods.ab.)).

This search strategy retrieved records that were indexed, rather than published, in February 2014. An information specialist (M. S.) ran a modified search strategy to retrieve records in each of the 3 mo prior to and following February 2014, which showed that the number of records entered into MEDLINE during February 2014 was representative of these other months.

### Screening

Screening was undertaken using online review software, DistillerSR. A form for screening of titles and abstracts (see S1 Forms) was used after being piloted on three records. Subsequently, three reviewers (M. J. P., L. S., and L. L.) screened all titles and abstracts using the method of liberal acceleration, whereby two reviewers needed to independently exclude a record for it to be excluded, while only one reviewer needed to include a record for it to be included. We retrieved the full text article for any citations meeting our eligibility criteria or for which eligibility remained unclear. A form for screening full text articles (see S1 Forms) was also piloted on three articles. Subsequently, two authors (of M. J. P., L. S., L. L., R. S.-O., E. K. R., and J. T.) independently screened each full text article. Any discrepancies in screening of titles/abstracts and full text articles were resolved via discussion, with adjudication by a third reviewer if necessary. In both these rounds of screening, articles were considered a SR if they met the Moher et al. [6] definition of a SR. Each full text article marked as eligible for inclusion was then screened a final time by one of two authors (M. J. P. or L. S.) to confirm that the article was consistent with the PRISMA-P 2015 definition of a SR (using the screening form in S1 Forms).

### Data Extraction and Verification

We performed data extraction on a random sample of 300 of the included SRs, which were selected using the random number generator in Microsoft Excel. We selected 300 SRs to match the number used in the 2004 sample. Sampling was stratified so that the proportion of Cochrane reviews in the selected sample equaled that in the total sample. Data were collected in DistillerSR using a standardized data extraction form including 88 items (see S1 Forms). The items were based on data collected in two previous studies [6,15] and included additional items to capture some issues not previously examined. All data extractors piloted the form on three SRs to ensure consistency in interpretation of data items. Subsequently, data from each SR were extracted by one of five reviewers (M. J. P., L. S., F. C.-L., R. S.-O., or E. K. R.). Data were extracted from both the article and any web-based appendices available.

At the end of data extraction, a 10% random sample of SRs (*n* = 30 SRs) was extracted independently in duplicate. Comparison of the data extracted revealed 42 items where a discrepancy existed between two reviewers on at least one occasion (items marked in S1 Forms). All discrepancies were resolved via discussion. To minimize errors in the remaining sample of SRs, one author (M. J. P.) verified the data for these 42 items in all SRs. Also, one author (M. J. P.) reviewed the free text responses of all items with an “Other (please specify)” option. Responses were modified if it was judged that one of the forced-choice options was a more appropriate selection.

### Data Analysis

All analyses were performed using Stata version 13 software [21]. Data were summarized as frequency and percentage for categorical items and median and interquartile range (IQR) for continuous items. We analyzed characteristics of all SRs and SRs categorized as Cochrane therapeutic (treatment/prevention), non-Cochrane therapeutic, epidemiology (e.g., prevalence, association between exposure and outcome), diagnosis/prognosis (e.g., diagnostic test accuracy, clinical prediction rules), or other (education, psychometric properties of scales, cost of illness). We anticipated that these different types of SRs would differ in the types of studies included (e.g., therapeutic SRs would more likely include randomized trials than epidemiology SRs). However, we considered nearly all of the other epidemiological and reporting characteristics as equally applicable to all types of SRs (i.e., all SRs, regardless of focus, should describe the methods of study identification, selection, appraisal, and synthesis). We have indicated in tables when a characteristic was not applicable (e.g., reporting of harms of interventions was only considered in therapeutic SRs). We also present, for all characteristics measured in the Moher et al. [6] sample, the percentage of SRs with each characteristic in 2004 compared with in 2014.

We explored whether the reporting of 26 characteristics of therapeutic SRs was associated with a SR being a Cochrane review and with self-reported use of the PRISMA Statement to guide conduct/reporting. The 26 characteristics were selected because they focused on whether a characteristic was reported or not (e.g., “Were eligible study designs stated?”) rather than on the detail provided (e.g., “Which study designs were eligible?”). We also explored whether the reporting of 15 characteristics of all SRs differed between the 2004 and 2014 samples (only 15 of the 26 characteristics were measured in both samples). Associations were quantified using the risk ratio, with 95% confidence intervals. The risk ratio was calculated because it is generally more interpretable than the odds ratio [22]. For the analysis of the association of reporting of characteristics with PRISMA use, we included only therapeutic SRs because PRISMA was designed primarily for this type of SR, and we excluded Cochrane SRs because they are supported by software that promotes good reporting.

The analyses described above were all prespecified before analyzing any data. The association between reporting and self-reported use of the PRISMA Statement was not included in our study protocol; it was planned following the completion of data extraction and prior to analysis. We planned to explore whether reporting characteristics of non-Cochrane SRs that were registered differed to non-Cochrane SRs that were unregistered, and whether reporting differed in SRs with a protocol compared to SRs without a protocol. However, there were too few SRs with a registration record (*n* = 12) or a SR protocol (*n* = 5) to permit reliable comparisons.

We performed a post hoc sensitivity analysis to see if the estimated prevalence of SRs was influenced by including articles that met the definition of a SR used by Moher et al. [6] but not the more explicit PRISMA-P 2015 definition.

## Results

### Search Results

**Fig 1:**
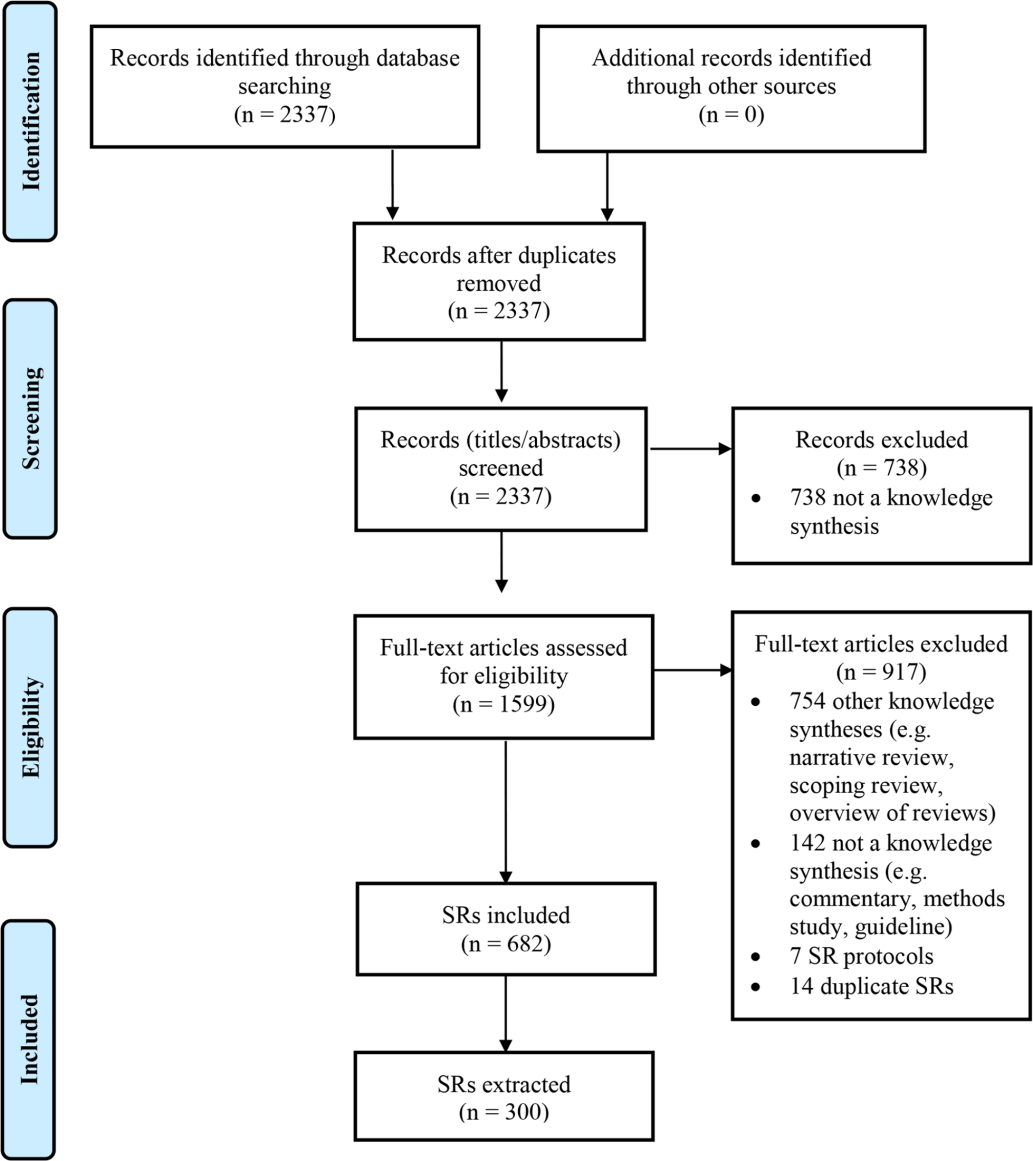
Flow diagram of identification, screening, and inclusion of SRs.

There were 2,337 records identified by the search (Fig 1). Screening of titles and abstracts led to the exclusion of 738 records. Of the 1,599 full text articles retrieved, 917 were excluded; most articles were not SRs but rather another type of knowledge synthesis (e.g., narrative review, scoping review, overview of reviews).

### Prevalence of SRs

We identified 682 SRs that were indexed in MEDLINE in February 2014. This figure suggests an annual publication rate of more than 8,000 SRs, which is equivalent to 22 SRs per day and is a 3-fold increase over what was observed in 2004 [6] (see calculations in S1 Results). One hundred (15%) of the SRs were Cochrane reviews. We identified 87 articles that were not counted in the final sample of 682 SRs but that would have met the less explicit definition of a SR used by Moher et al. [6] for the 2004 sample. In a sensitivity analysis, adding these to the final sample raised the SR prevalence to 769 SRs indexed in the month of February 2014, which is equivalent to more than 9,000 SRs per year and 25 SRs per day being indexed in MEDLINE.

### Epidemiological Characteristics of SRs

Data were collected on the random sample of 45 Cochrane and 255 non-Cochrane SRs (Table 1). The 300 SRs were published in 185 journals, most of which published only one SR during the month (141/185 [76%]) and had an impact factor less than 5.0 (187/300 [62%]). Only 5/300 (2%) SRs were published in journals with a high impact factor (i.e., Thomson ISI Journal Impact Factor 2012 > 15.0). Most of the SRs (250/300 [83%]) were published in the latter half of 2013. The median number of authors was five (IQR 4–6), and only 5/300 (2%) SRs were conducted by one author. The corresponding authors were based most commonly in China, the UK, or the US; corresponding authors from these three countries were responsible for 155/300 (52%) of the examined SRs. Just over half of the SRs (164/300 [55%]) were classified as therapeutic, 74/300 (25%) as epidemiology, 33/300 (11%) as diagnosis/prognosis, and 29/300 (10%) as other. All Cochrane SRs focused on a therapeutic question. There was wide diversity in the clinical conditions investigated; 20 ICD-10 codes were recorded across the SRs, with neoplasms, infections and parasitic diseases, and diseases of the circulatory system the most common (each investigated in >10% of SRs). All SRs were written in English.

**Table 1:** Epidemiology of 300 systematic reviews indexed in February 2014.

Characteristic	Category	Number (Percent)^a^
**Total number of journals**		185
**Number of SRs per journal**	1	141/185 (76%)
	2	30/185 (16%)
	3	8/185 (4%)
	≥4	6/185 (3%)
**Journal impact factor (2012)**	0.0–5.0	187 (62%)
	5.1–10.0	74 (25%)
	10.1–15.0	4 (1%)
	>15.0	5 (2%)
	No impact factor	30 (10%)
**Year of publication**	2014	42 (14%)
	2013	250 (83%)
	2012	7 (2%)
	2007	1 (1%)
**Number of authors**	1	5 (2%)
	2–3	64 (21%)
	4–6	162 (54%)
	≥7	69 (23%)
**Country of corresponding author**	China	62 (21%)
	UK	47 (16%)
	US	46 (15%)
	Canada	27 (9%)
	The Netherlands	18 (6%)
	Australia	16 (5%)
	Germany	10 (3%)
	Other (<10 reviews/country, 30 countries)	74 (25%)
**Focus of review**	Therapeutic (treatment/prevention)	164 (55%)
	Epidemiology (prevalence, associations/etiology)	74 (25%)
	Diagnosis	12 (4%)
	Prognosis	21 (7%)
	Other (e.g., education, review of psychometric properties, barriers analysis, cost of illness)	29 (10%)
**Common ICD-10 codes**	Neoplasms (including cancers, carcinomas, tumors)	49 (16%)
	Infections and parasitic diseases	41 (14%)
	Diseases of the circulatory system	34 (11%)
	Diseases of the digestive system	25 (8%)
	Endocrine, nutritional, and metabolic diseases	23 (8%)
	Mental and behaviour disorders	22 (7%)
	Diseases of the musculoskeletal system	20 (7%)
**Cochrane review**		45 (15%)

^a^Illustrative binomial 95% confidence intervals for percentages when sample size is 300: 1% (0.2% to 3%); 5% (3% to 8%); 10% (7% to 14%); 25% (20% to 30%); 50% (44% to 56%); 75% (70% to 80%).

Most SRs (263/300 [88%]) were published in specialty journals (Table 2). Only 31/300 (10%) were updates of a previous SR. The majority of these were Cochrane SRs (25/31 [81%]); only one diagnosis/prognosis SR and no epidemiology SRs were described as an update. Of the therapeutic SRs, 76/164 (46%) investigated a pharmacological intervention, 75/164 (46%) investigated a non-pharmacological intervention, and 13/164 (8%) investigated both types of intervention. A median of 15 studies involving 2,072 participants were included in the SRs overall, but the number of studies and participants varied according to the type of SR. Cochrane SRs included fewer studies (median 9 versus 14 in non-Cochrane therapeutic SRs), and epidemiology SRs included a larger number of participants (median 8,154 versus 1,449 in non-epidemiology SRs). Only 4/300 (1%) SRs were “empty reviews” (i.e., identified no eligible studies). Meta-analysis was performed in 189/300 (63%) SRs, with a median of 9 (IQR 6–17) studies included in the largest meta-analysis in each SR that included one or more meta-analyses. Harms of interventions were collected (or planned to be collected) in 113/164 (69%) therapeutic SRs. Few SRs (23/172 [13%]) considered costs associated with interventions or illness.

**Table 2:** Epidemiology of systematic reviews indexed in February 2014, subgrouped by focus of SR.

Characteristic	Type of SR					
	All (*n* = 300)	Therapeutic (Cochrane) (*n* = 45)	Therapeutic (Non-Cochrane) (*n* = 119)	Epidemiology (*n* = 74)	Diagnosis/Prognosis (*n* = 33)	Other (*n* = 29)
**Journal type**						
General	37 (12%)	0 (0%)	21 (18%)	10 (14%)	4 (12%)	2 (7%)
Specialty	263 (88%)	45 (100%)	98 (82%)	64 (86%)	29 (88%)	27 (93%)
**Number of authors**	5 (4–6)	4 (3–6)	5 (4–6)	5 (4–6)	5 (4–7)	4 (3–5)
**Update of a previous SR**	31 (10%)	25 (56%)	5 (4%)	0 (0%)	1 (3%)	0 (0%)
**Types of interventions**						
Pharmacological	76/164 (46%)	23 (51%)	53 (45%)	NA	NA	NA
Non-pharmacological	75/164 (46%)	17 (38%)	58 (49%)	NA	NA	NA
Both	13/164 (8%)	5 (11%)	8 (7%)	NA	NA	NA
**Number of included studies**	15 (8–25)	9 (4–17)	14 (8–23)	17 (11–29)	15 (9–25)	18 (12–30)
**Number of included participants** ^**a**^	2,072 (672–8,033)	1,113 (421–2,751)	1,565 (509–4,677)	8,154 (2,752–124,489)	1,436 (898–3,060)	805 (517–4,630)
**Empty review (no eligible studies)**	4 (1%)	3 (7%)	1 (1%)	0 (0%)	0 (0%)	0 (0%)
**Meta-analysis performed**	189 (63%)	32 (71%)	78 (66%)	49 (66%)	25 (76%)	5 (17%)
**Number of studies included in the largest meta-analysis in each SR that included meta-analysis**	9 (6–17)	6 (3–11)	8 (5–15)	10 (7–19)	11 (8–19)	26 (17–28)
**Harms considered** ^**b**^	113/164 (69%)	41 (91%)	72 (61%)	NA	NA	NA
**Economics (i.e., costs) considered** ^**b**^	23/172 (13%)	7 (16%)	8 (7%)	NA	NA	8/29 (28%)

Data given as number (percent) or median (IQR). The denominator of fractions indicates the number of reports where the variable concerned was considered relevant to the SR. Illustrative binomial 95% confidence intervals for percentages when sample size is 300: 1% (0.2% to 3%); 5% (3% to 8%); 10% (7% to 14%); 25% (20% to 30%); 50% (44% to 56%); 75% (70% to 80%).

^a^The total number of included participants was reported (or able to be calculated) in only 247 SRs overall, 44 Cochrane therapeutic SRs, 99 non-Cochrane therapeutic SRs, 60 epidemiology SRs, 32 diagnosis/prognosis SRs, and 12 other SRs.

^b^“Considered” means that either data for the outcome were reported or the authors planned to collect data for the outcome if such data were reported in the included studies.

NA, not applicable.

### Reporting Characteristics of SRs

Here we summarize a subset of the characteristics of the SRs about which data were collected in this study (Table 3; data for all items are presented in S1 Results). Many SRs (254/300 [85%]) included the term “systematic review” or “meta-analysis” in the title or abstract. This percentage increased to 94% (239/255) when Cochrane SRs, which generally do not include these terms in their title, were omitted. A few SRs (12/300 [4%]) had been prospectively registered (e.g., in PROSPERO). In rather more SRs, authors mentioned working from a review protocol (77/300 [26%]), but a publicly accessible protocol was cited in only 49/300 (16%). These figures were driven almost entirely by Cochrane SRs; a publicly accessible protocol was cited in only 5/119 (4%) non-Cochrane therapeutic SRs and in no epidemiology, diagnosis/prognosis, or other SRs. Authors reported using a reporting guideline (e.g., PRISMA [7], MOOSE [23]) in 87/300 (29%) SRs. The purpose of these guidelines was frequently misinterpreted; in 45/87 (52%) of these SRs, it was stated that the reporting guideline was used to guide the conduct, not the reporting, of the SR (S1 Results). In 93/255 (36%) non-Cochrane SRs, authors reported using Cochrane methods (e.g., cited the *Cochrane Handbook for Systematic Reviews of Interventions* [19]).

**Table 3:** Reporting characteristics of systematic reviews indexed in February 2014 sample.

Category	Characteristic	Type of SR					
		All (*n* = 300)	Therapeutic (Cochrane) (*n* = 45)	Therapeutic (Non-Cochrane) (*n* = 119)	Epidemiology (*n* = 74)	Diagnosis/Prognosis (*n* = 33)	Other (*n* = 29)
**Administrative information**	**“Systematic review” or “meta-analysis” used in title/abstract**	254 (85%)	15 (33%)	113 (95%)	69 (93%)	32 (97%)	25 (86%)
	**SR registration (e.g., PROSPERO) mentioned**	12 (4%)	0 (0%)	8 (7%)	3 (4%)	1 (3%)	0 (0%)
	**SR protocol mentioned**						
	Protocol is publicly available	49 (16%)	44 (98%)	5 (4%)	0 (0%)	0 (0%)	0 (0%)
	Protocol mentioned, but not publicly available	28 (9%)	0 (0%)	21 (18%)	3 (4%)	2 (6%)	2 (7%)
	**Reporting guideline (e.g., PRISMA) mentioned**	87 (29%)	1 (2%)	42 (35%)	27 (36%)	8 (24%)	9 (31%)
	**Cochrane methods used**	138 (46%)	45 (100%)	64 (54%)	16 (22%)	9 (27%)	4 (14%)
**Study eligibility criteria**	**Eligible publication status**						
	Both published and unpublished studies	116 (39%)	41 (91%)	49 (41%)	13 (18%)	6 (18%)	7 (24%)
	Only published studies	80 (27%)	2 (4%)	33 (28%)	25 (34%)	9 (27%)	11 (38%)
	Only unpublished studies	1 (1%)	0 (0%)	1 (1%)	0 (0%)	0 (0%)	0 (0%)
	Not reported	103 (34%)	2 (4%)	36 (30%)	36 (49%)	18 (55%)	11 (38%)
	**Eligible languages**						
	All languages considered	129 (43%)	37 (82%)	48 (40%)	29 (39%)	10 (30%)	5 (17%)
	English only	92 (31%)	1 (2%)	44 (37%)	22 (30%)	12 (36%)	13 (45%)
	Mixed (English and a specific LOE)	31 (10%)	1 (2%)	9 (8%)	10 (14%)	5 (15%)	6 (21%)
	Only LOE	0 (0%)	0 (0%)	0 (0%)	0 (0%)	0 (0%)	0 (0%)
	Not reported	48 (16%)	6 (13%)	18 (15%)	13 (18%)	6 (18%)	5 (17%)
	**Eligibility/ineligibility criteria based on study designs reported**	237 (79%)	45 (100%)	104 (87%)	56 (77%)	17 (52%)	15 (52%)
	**Eligible study designs**						
	RCTs	158 (53%)	44 (98%)	99 (83%)	7 (9%)	1 (3%)	7 (24%)
	Quasi-RCTs	33 (11%)	14 (31%)	15 (13%)	3 (4%)	0 (0%)	1 (3%)
	Other controlled experimental studies (e.g., non-randomized controlled trials, controlled before-and-after studies, interrupted time series studies)	30 (10%)	4 (9%)	18 (15%)	5 (7%)	1 (3%)	2 (7%)
	Observational—cohort studies	76 (25%)	0 (0%)	25 (21%)	37 (50%)	10 (30%)	4 (14%)
	Observational—case–control studies	49 (16%)	0 (0%)	8 (7%)	37 (50%)	2 (6%)	2 (7%)
	Observational—cross-sectional studies	31 (10%)	0 (0%)	10 (8%)	17 (23%)	0 (0%)	4 (14%)
	Observational—case studies or case series	19 (6%)	0 (0%)	10 (8%)	6 (8%)	2 (6%)	1 (3%)
	Other (e.g., observational studies of unspecified type, qualitative studies)	56 (19%)	1 (2%)	22 (18%)	11 (15%)	8 (24%)	14 (48%)
	Unclear/not stated	36 (12%)	0 (0%)	3 (3%)	7 (9%)	16 (48%)	10 (34%)
	Restricted to RCTs and quasi-RCTs	107 (36%)	40 (89%)	64 (54%)	1 (1%)	1 (3%)	1 (3%)
**Search methods**	**Number of databases searched**	4 (3–5)	5 (4–6)	3 (2–5)	3 (2–5)	3 (2–3)	5 (3–6)
	**Only one database searched**	28 (9%)	0 (0%)	11 (9%)	13 (18%)	1 (3%)	3 (10%)
	**Years of coverage reported**						
	Both start and end dates are reported for all databases	196 (65%)	41 (91%)	78 (66%)	39 (53%)	19 (58%)	19 (66%)
	Partially—start and end dates are reported for one of many databases, or only the end date is reported for all databases	88 (29%)	4 (9%)	35 (29%)	30 (41%)	13 (39%)	6 (21%)
	**Search terms reported**						
	Full Boolean search logic reported for one or more databases	134 (45%)	44 (98%)	41 (34%)	26 (35%)	13 (39%)	10 (34%)
	Main index terms (e.g., MeSH) reported	36 (12%)	0 (0%)	17 (14%)	11 (15%)	5 (15%)	3 (10%)
	Free text words reported	138 (46%)	0 (0%)	65 (55%)	41 (55%)	18 (55%)	14 (48%)
	No search terms reported	16 (5%)	1 (2%)	7 (6%)	3 (4%)	1 (3%)	4 (14%)
	**Trial registry (e.g., ClinicalTrials.gov) searched**	58 (19%)	28 (62%)	24 (20%)	4 (5%)	2 (6%)	0 (0%)
	**Number of other sources searched**	1 (1–2)	2 (1–3)	1 (1–2)	1 (1–1)	1 (1–1)	1 (1–2)
	**Other sources searched**						
	Searched grey literature database (e.g., OpenSIGLE)	21 (7%)	9 (20%)	8 (7%)	1 (1%)	2 (6%)	1 (3%)
	Reviewed reference lists of relevant studies, reviews, or textbooks	243 (81%)	38 (84%)	99 (83%)	58 (78%)	27 (82%)	21 (72%)
	Hand searched particular journal(s)	25 (8%)	6 (13%)	12 (10%)	2 (3%)	1 (3%)	4 (14%)
	Reviewed abstracts/proceedings of specific conference(s)	47 (16%)	11 (24%)	26 (22%)	7 (9%)	1 (3%)	2 (7%)
	Contacted experts or corresponding authors of included studies	54 (18%)	23 (51%)	16 (13%)	8 (11%)	5 (15%)	2 (7%)
	Contacted a drug or device manufacturer	11 (4%)	8 (18%)	3 (3%)	0 (0%)	0 (0%)	0 (0%)
	Contacted a drug or device regulator (e.g., US Food and Drug Administration, European Medicines Agency)	2 (1%)	0 (0%)	2 (2%)	0 (0%)	0 (0%)	0 (0%)
	Other (e.g., citation tracking, personal files)	35 (12%)	8 (18%)	13 (11%)	5 (7%)	6 (18%)	3 (10%)
**Screening, extraction, and risk of bias assessment methods**	**Screening method**						
	All identified studies screened by at least two authors	200 (67%)	44 (98%)	73 (61%)	41 (55%)	24 (73%)	18 (62%)
	All identified studies screened by one author, with a sample screened by another	7 (2%)	1 (2%)	3 (3%)	3 (4%)	0 (0%)	0 (0%)
	All identified studies screened by only one author	6 (2%)	0 (0%)	3 (3%)	1 (1%)	0 (0%)	2 (7%)
	Not reported	87 (29%)	0 (0%)	40 (34%)	29 (39%)	9 (27%)	9 (31%)
	**Data extraction method**						
	All data extracted by at least two authors	163/296 (55%)	41/42 (98%)	49/118 (42%)	43/74 (58%)	22/33 (67%)	8/29 (28%)
	All data extracted by one author, with verification by another	29/296 (10%)	0 (0%)	15/118 (13%)	9/74 (12%)	1/33 (3%)	4/29 (14%)
	All data extracted by only one author	7/296 (2%)	0 (0%)	2/118 (2%)	0 (0%)	2/33 (6%)	3/29 (10%)
	Not reported	97/296 (33%)	1/42 (2%)	52/118 (44%)	22/74 (30%)	8/33 (24%)	14/29 (48%)
	**Study risk of bias/quality formally assessed**	206/296 (70%)	42/42 (100%)	87/118 (74%)	44/74 (59%)	22/33 (67%)	11/29 (38%)
	**Study risk of bias/quality assessment method**						
	All included studies assessed by at least two authors	121/206 (59%)	37/42 (88%)	41/87 (47%)	24/44 (55%)	14/22 (64%)	5/11 (45%)
	All included studies assessed by one author, with verification by another	6/206 (3%)	0 (0%)	4/87 (5%)	2/44 (5%)	0 (0%)	0 (0%)
	All included studies assessed by only one author	3/206 (1%)	0 (0%)	1/87 (1%)	1/44 (2%)	0 (0%)	1/11 (9%)
	Not reported	76/206 (37%)	5/42 (12%)	41/87 (47%)	17/44 (39%)	8/22 (36%)	5/11 (45%)
	**Study risk of bias/quality assessment tool used**						
	Cochrane risk of bias tool (or modification)	77/206 (37%)	37/42 (88%)	36/87 (41%)	4/44 (9%)	0 (0%)	0 (0%)
	Jadad scale (or modification)	17/206 (8%)	0 (0%)	17/87 (20%)	0 (0%)	0 (0%)	0 (0%)
	Newcastle-Ottawa Scale (or modification)	17/206 (8%)	1/42 (2%)	6/87 (7%)	8/44 (18%)	1/22 (5%)	1/11 (9%)
	QUADAS or QUADAS-2	8/206 (4%)	0 (0%)	0 (0%)	0 (0%)	7/22 (32%)	1/11 (9%)
	Reporting guideline (e.g., CONSORT)	15/206 (7%)	0 (0%)	9/87 (10%)	3/44 (7%)	2/22 (9%)	1/11 (9%)
	Tool developed by review authors	38/206 (18%)	2/42 (5%)	9/87 (10%)	16/44 (36%)	8/22 (36%)	3/11 (27%)
	Other	53/206 (26%)	3/42 (7%)	21/87 (24%)	14/44 (32%)	8/22 (36%)	7/11 (64%)
	Not reported	6/206 (3%)	0 (0%)	2/87 (2%)	2/44 (5%)	2/22 (9%)	0 (0%)
	**Study risk of bias/quality assessment incorporated into meta-analysis**	31/189 (16%)	4/32 (13%)	11/78 (14%)	10/49 (20%)	4/25 (16%)	2/5 (40%)
	**Selective reporting assessed**	70/296 (24%)	36/42 (86%)	30/118 (25%)	3/74 (4%)	1/33 (30%)	0 (0%)
**Included/excluded studies and participants**	**Review flow reported**						
	Completely in PRISMA-like flow diagram	206 (69%)	23 (51%)	94 (79%)	46 (62%)	26 (79%)	17 (59%)
	Completely in text/table only	20 (7%)	5 (11%)	5 (4%)	6 (8%)	2 (6%)	2 (7%)
	Partially reported	38 (13%)	7 (16%)	14 (12%)	11 (15%)	2 (6%)	4 (14%)
	Not reported	36 (12%)	10 (22%)	6 (5%)	11 (15%)	3 (9%)	6 (21%)
	**Reasons for exclusion of full text articles reported**						
	Reasons for all excluded articles reported in PRISMA-like flow diagram or text/table	211 (70%)	41 (91%)	87 (73%)	42 (57%)	28 (85%)	13 (45%)
	Partially—reasons for only some excluded articles reported	28 (9%)	4 (9%)	9 (8%)	13 (18%)	1 (3%)	1 (3%)
	Not reported for any articles	61 (20%)	0 (0%)	23 (19%)	19 (26%)	4 (12%)	15 (52%)
	**Grey literature (e.g., conference abstracts) included**	26 (9%)	8 (18%)	11 (9%)	2 (3%)	1 (3%)	4 (14%)
	**Total number of included participants reported**						
	In main text	194/296 (66%)	39/42 (93%)	83/118 (70%)	42/74 (57%)	26/33 (79%)	4/29 (14%)
	In abstract	147/296 (50%)	37/42 (88%)	56/118 (47%)	34/74 (46%)	17/33 (52%)	3/29 (10%)
**Outcomes**	**At least one outcome stated in methods**	234 (78%)	45 (100%)	99 (83%)	49 (66%)	28 (85%)	13 (45%)
	**Number of outcomes stated**	4 (2–6)	6 (5–9)	4 (2–6)	1 (1–3)	2 (2–4)	4 (2–5)
	**Primary outcome stated**	136/288 (47%)	43 (96%)	56 (47%)	30 (41%)	6/21 (29%)	1 (3%)
	**Type of primary outcome**						
	Dichotomous	91/136 (67%)	27/43 (63%)	36/56 (64%)	25/30 (83%)	3/6 (50%)	0 (0%)
	Continuous	29/136 (21%)	10/43 (23%)	16/56 (29%)	2/30 (7%)	0 (0%)	1/1 (100%)
	Rate	5/136 (4%)	2/43 (5%)	1/56 (2%)	2/30 (7%)	0 (0%)	0 (0%)
	Time-to-event	9/136 (7%)	4/43 (9%)	2/56 (4%)	0 (0%)	3/6 (50%)	0 (0%)
	Other (e.g., prevalence, not specified)	2/136 (1%)	0 (0%)	1/56 (2%)	1/30 (3%)	0 (0%)	0 (0%)
	**Statistical significance of intervention effect estimate for primary outcome**						
	Favourable, statistically significant	53/88 (60%)	18/36 (50%)	35/52 (67%)	NA	NA	NA
	Favourable, statistically non-significant	23/88 (26%)	12/36 (33%)	11/52 (21%)	NA	NA	NA
	Unfavourable, statistically significant	0 (0%)	0 (0%)	0 (0%)	NA	NA	NA
	Unfavourable, statistically non-significant	9/88 (10%)	6/36 (17%)	3/52 (6%)	NA	NA	NA
	Direction of effect unclear	3/88 (3%)	0 (0%)	3/52 (6%)	NA	NA	NA
**Statistical methods**	**Meta-analysis performed**	189 (63%)	32 (71%)	78 (66%)	49 (66%)	25 (76%)	5 (17%)
	**Meta-analysis model used**						
	Fixed-effects model for all meta-analyses	34/189 (18%)	14/32 (44%)	12/78 (15%)	4/49 (8%)	2/25 (8%)	2/5 (40%)
	Random-effects model for all meta-analyses	89/189 (47%)	7/32 (22%)	40/78 (51%)	25/49 (51%)	15/25 (60%)	2/5 (40%)
	Varied across meta-analyses	54/189 (29%)	11/32 (34%)	21/78 (27%)	16/49 (33%)	6/25 (24%)	0 (0%)
	Not reported	12/189 (6%)	0 (0%)	5/78 (6%)	4/49 (8%)	2/25 (8%)	1/5 (20%)
	**Statistical heterogeneity investigated**						
	Using statistical methods or qualitatively assessed (e.g., via narrative discussion)	207/300 (69%)	33/45 (73%)	86/119 (72%)	52/74 (70%)	28/33 (85%)	8/29 (28%)
	Using statistical methods when meta-analysis performed	175/189 (93%)	32/32 (100%)	71/78 (91%)	45/49 (92%)	24/25 (96%)	3/5 (60%)
	**Heterogeneity statistic inappropriately guided choice of meta-analysis model** (**e.g., random-effects model selected if *I*** ^**2**^ **> 50%)**	72/189 (38%)	8/32 (25%)	27/78 (35%)	22/49 (45%)	15/25 (60%)	0 (0%)
	**Risk of publication bias assessed (or intent to assess)**						
	Formally assessed (e.g., funnel plot, sensitivity analysis)	93 (31%)	7 (16%)	39 (33%)	33 (45%)	13 (39%)	1 (3%)
	Not assessed, but authors planned to if they identified a sufficient number of studies	37 (12%)	28 (62%)	7 (6%)	0 (0%)	2 (6%)	0 (0%)
	**Possibility of publication bias discussed/considered in results, discussion, or conclusion**	141 (47%)	29 (64%)	55 (46%)	37 (50%)	18 (55%)	2 (7%)
	**Additional analyses**						
	Subgroup analysis	87/189 (46%)	12/32 (38%)	40/78 (51%)	23/49 (47%)	8/25 (32%)	4/5 (80%)
	Sensitivity analysis	92/189 (49%)	12/32 (38%)	43/78 (55%)	27/49 (55%)	9/25 (36%)	1/5 (20%)
	Meta-regression	21/189 (11%)	0 (0%)	7/78 (9%)	10/49 (20%)	2/25 (8%)	2/5 (40%)
	Network meta-analysis	7 (2%)	0 (0%)	7 (6%)	0 (0%)	0 (0%)	0 (0%)
	Individual participant data meta-analysis	2 (1%)	2 (4%)	0 (0%)	0 (0%)	0 (0%)	0 (0%)
	Other (cumulative meta-analysis, multivariate meta-analysis, trial sequential analysis, unweighted pooling)	11 (4%)	1 (2%)	3 (3%)	5 (7%)	1 (3%)	1 (3%)
**Limitations, conclusions, COIs, and funding**	**GRADE assessment reported in a summary of findings table or text**	32 (11%)	27 (60%)	4 (3%)	0 (0%)	1 (3%)	0 (0%)
	**Limitations reported**						
	Both limitations at study level and review level reported	173 (58%)	32 (71%)	63 (53%)	43 (58%)	23 (70%)	12 (41%)
	Only limitations at study level reported	67 (22%)	10 (22%)	31 (26%)	15 (20%)	5 (15%)	6 (21%)
	Only limitations at review level reported	27 (9%)	0 (0%)	10 (8%)	8 (11%)	3 (9%)	6 (21%)
	**Study risk of bias/quality/limitations incorporated into therapeutic SR abstract conclusions**	99/164 (60%)	42/45 (93%)	57/119 (48%)	NA	NA	NA
	**COIs reported**						
	COIs of SR authors reported	260 (87%)	45 (100%)	103 (87%)	61 (82%)	30 (91%)	21 (72%)
	COIs or funding of authors of included studies reported	21/296 (7%)	13/42 (31%)	7/118 (6%)	0 (0%)	0 (0%)	1 (3%)
	**Source of funding of the SR**						
	Non-profit	142 (47%)	38 (84%)	48 (40%)	30 (41%)	9 (27%)	17 (59%)
	For-profit	8 (3%)	0 (0%)	3 (3%)	2 (3%)	0 (0%)	3 (10%)
	Mixed	2 (1%)	0 (0%)	1 (1%)	1 (1%)	0 (0%)	0 (0%)
	Authors specified there was no funding	39 (13%)	5 (11%)	14 (12%)	11 (15%)	7 (21%)	2 (7%)
	Not reported	109 (36%)	2 (4%)	53 (45%)	30 (41%)	17 (51%)	7 (24%)

Data given as number (percent) or median (IQR). The denominator of fractions indicates the number of reports where the variable concerned was considered relevant to the SR. Illustrative binomial 95% confidence intervals for percentages when sample size is 300: 1% (0.2% to 3%); 5% (3% to 8%); 10% (7% to 14%); 25% (20% to 30%); 50% (44% to 56%); 75% (70% to 80%).

COI, conflict of interest; LOE, language other than English; NA, not applicable; RCT, randomized controlled trial.

### Reporting of Study Eligibility Criteria

At least one eligibility criterion was reported in the majority of SRs, but there was wide variation in the content and quality of reporting. In 116/300 (39%) SRs, authors specified that both published and unpublished studies were eligible for inclusion, while a quarter restricted inclusion to published studies (80/300 [27%]). However, in 103/300 (34%) SRs, publication status criteria were not reported. Language criteria were reported in 252/300 (84%) SRs, with more SRs considering all languages (129/300 [43%]) than considering English only (92/300 [31%]). Study design inclusion criteria were stated in 237/300 (79%) SRs. Nearly all Cochrane SRs (40/45 [89%]) restricted inclusion to randomized or quasi-randomized controlled trials, whereas only 64/119 (54%) non-Cochrane therapeutic SRs did so. Epidemiology, diagnosis/prognosis, and other SRs included a range of study designs, mostly observational (e.g., cohort, case–control).

### Reporting of Search Methods

A median of four electronic bibliographic databases (IQR 3–5) were searched by review authors. Only 28/300 (9%) SRs searched only one database, and nearly all of these were non-Cochrane therapeutic (*n* = 11) or epidemiology (*n* = 13) SRs. Years of coverage of the search were completely reported in 196/300 (65%) SRs, and a full Boolean search logic for at least one database was reported in 134/300 (45%). Authors searched a median of one (IQR 1–2) other source, of which reviewing the references lists of included studies was described most often (243/300 [81%]). Sources of unpublished data were infrequently searched, for example, grey literature databases such as OpenSIGLE (21/300 [7%]) or drug or device regulator databases such as Drugs@FDA (2/164 therapeutic SRs [1%]). Searching of trial registries (e.g., ClinicalTrials.gov) was reported in 58/300 (19%) SRs, more often in Cochrane therapeutic SRs (28/45 [62%]) than in non-Cochrane therapeutic SRs (24/119 [20%]).

### Reporting of Screening, Data Extraction, and Risk of Bias Assessment Methods

There was no information in approximately a third of SRs on how authors performed screening (87/300 [29%]), data extraction (97/296 [33%]), or risk of bias assessment (76/206 [37%]) (i.e., number of SR authors performing each task, whether tasks were conducted independently by two authors or by one author with verification by another). Of the SRs that reported this information, very few (<4%) stated that only one author was responsible for these tasks. Risk of bias/quality assessment was formally conducted in 206/296 (70%) SRs, more often in therapeutic SRs (129/160 [81%]) than in epidemiology, diagnosis/prognosis, or other SRs (77/136 [57%]). Many different assessment tools were used. The Cochrane risk of bias tool for randomized trials [11] was the most commonly used tool in both Cochrane (37/42 [88%]) and non-Cochrane therapeutic SRs (36/87 [41%]). Author-developed tools were used in 38/206 (18%) SRs overall and were used more commonly than validated tools in epidemiology SRs (16/44 [36%] used a tool developed by the review authors, while 8/44 [18%] used the Newcastle–Ottawa Scale [24]). Risk of bias information was incorporated into the analysis (e.g., via subgroup or sensitivity analyses) in only 31/189 (16%) SRs with meta-analysis.

### Reporting of Included/Excluded Studies and Participants

A review flow was reported in the majority of SRs (226/300 [75%]) and was most often presented in a PRISMA/QUOROM-like flow diagram (206/300 [69%]). Reasons for excluding studies were described in 211/300 (70%) SRs overall but were described less often in epidemiology (42/74 [57%]) and other (13/29 [45%]) SRs. At least one type of grey literature (e.g., conference abstract, thesis) was stated to have been included in 26/300 (9%) SRs. In many SRs, it was difficult to discern the total number of participants across the included studies, as this figure was described in the full text of only 194/296 (66%) SRs and in the abstract of only 147/296 (50%) SRs. Such information was infrequently reported in the SRs classified as other (4/29 [14%] in the full text and 3/29 [10%] in the abstract).

### Reporting of Review Outcomes

Authors collected data on a median of four (IQR 2–6) outcomes; however, no outcomes were specified in the methods section of 66/300 (22%) SRs. An outcome was described in the SR as “primary” in less than half of the SRs (136/288 [47%]). Given that we did not seek SR protocols, it is unclear how often the primary outcome was selected a priori. Most primary outcomes were dichotomous (91/136 [67%]). Of the 99 therapeutic SRs with a primary outcome, a *p*-value or 95% confidence interval was reported for 88/99 (89%) of the primary outcome intervention effect estimates. Of these estimates, 53/88 (60%) were statistically significant and favourable to the intervention, while none were statistically significantly unfavourable to the intervention.

### Reporting of Statistical Methods

Meta-analysis was performed in 189/300 (63%) SRs. The random-effects model was used more often than the fixed-effects model (89/189 [47%] versus 34/189 [18%]), and statistical heterogeneity was examined in nearly all of these SRs (175/189 [93%]). In a third of SRs (72/189 [38%]), it was stated that a heterogeneity statistic guided the choice of the meta-analysis model (e.g., random-effects model used if *I* ^2^ > 50%). Methods commonly used to infer publication bias (e.g., funnel plot, test for small-study effects) were used in approximately a third of SRs (93/300 [31%]). Of these, only 53/93 (57%) SRs had a sufficient number of studies for the analysis (i.e., a meta-analysis that included at least ten studies [25]). It would have been inappropriate to use such methods in 169/300 (56%) SRs due to the lack of meta-analysis or insufficient number of studies. Authors discussed the possibility of publication bias (regardless of whether it was formally assessed) in 141/300 (47%) SRs. In just under half of the SRs with meta-analysis, authors reported subgroup analyses (87/189 [46%]) or sensitivity analyses (92/189 [49%]). Advanced meta-analytic techniques were rarely used, for example, network meta-analysis (7/300 [2%]) and individual participant data meta-analysis (2/300 [1%]).

### Reporting of Limitations, Conclusions, Conflicts of Interest, and Funding

Few SRs included a GRADE assessment of the quality of the body of evidence (32/300 [11%]); nearly all of those that did were Cochrane SRs (27/32 [84%]). Limitations of either the review or included studies (or both) were stated in nearly all SRs (267/300 [89%]), although in 67/300 (22%) no review limitations were considered. Study limitations (including risk of bias/quality) were incorporated into the abstract conclusions of only 99/164 (60%) therapeutic SRs. Most review authors declared whether they had any conflicts of interest (260/300 [87%]). In contrast, only 21/296 (7%) reported the conflicts of interest or funding sources of the studies included in their SR. Almost half of the SRs were funded by a non-profit source (142/300 [47%]), while only 8/300 (3%) were clearly funded by for-profit sources. However, the funding source was not declared in a third of SRs (109/300 [36%]); this non-reporting was more common in non-Cochrane therapeutic (53/119 [45%]), epidemiology (30/74 [41%]), and diagnosis/prognosis SRs (17/33 [51%]) than in Cochrane therapeutic SRs (2/45 [4%]).

### Influence of Cochrane Status and Self-Reported Use of PRISMA on Reporting Characteristics

**Fig 2:**
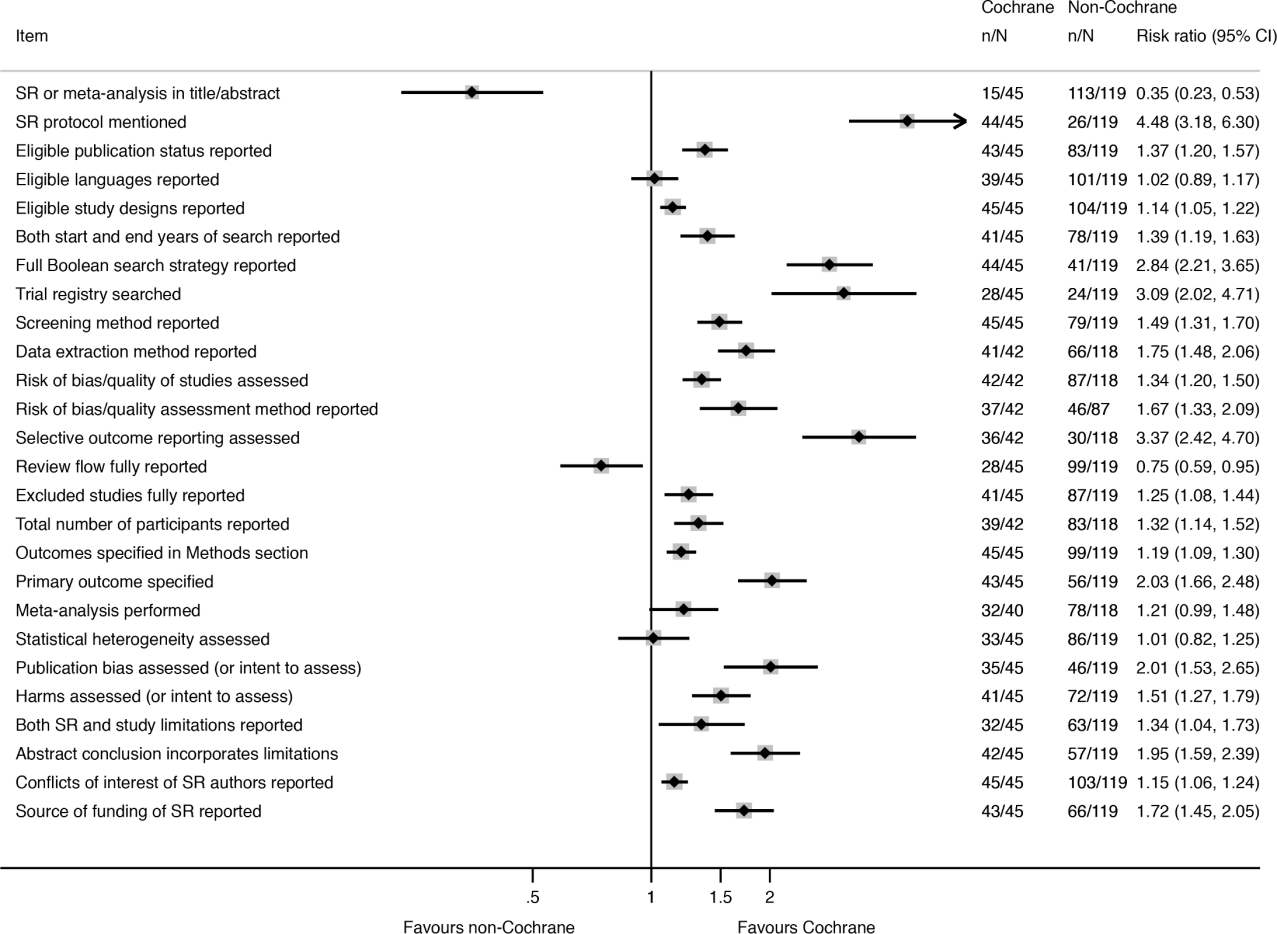
Unadjusted risk ratio associations between reporting characteristics and type of SR: Cochrane versus non-Cochrane therapeutic SRs.

**Fig 3:**
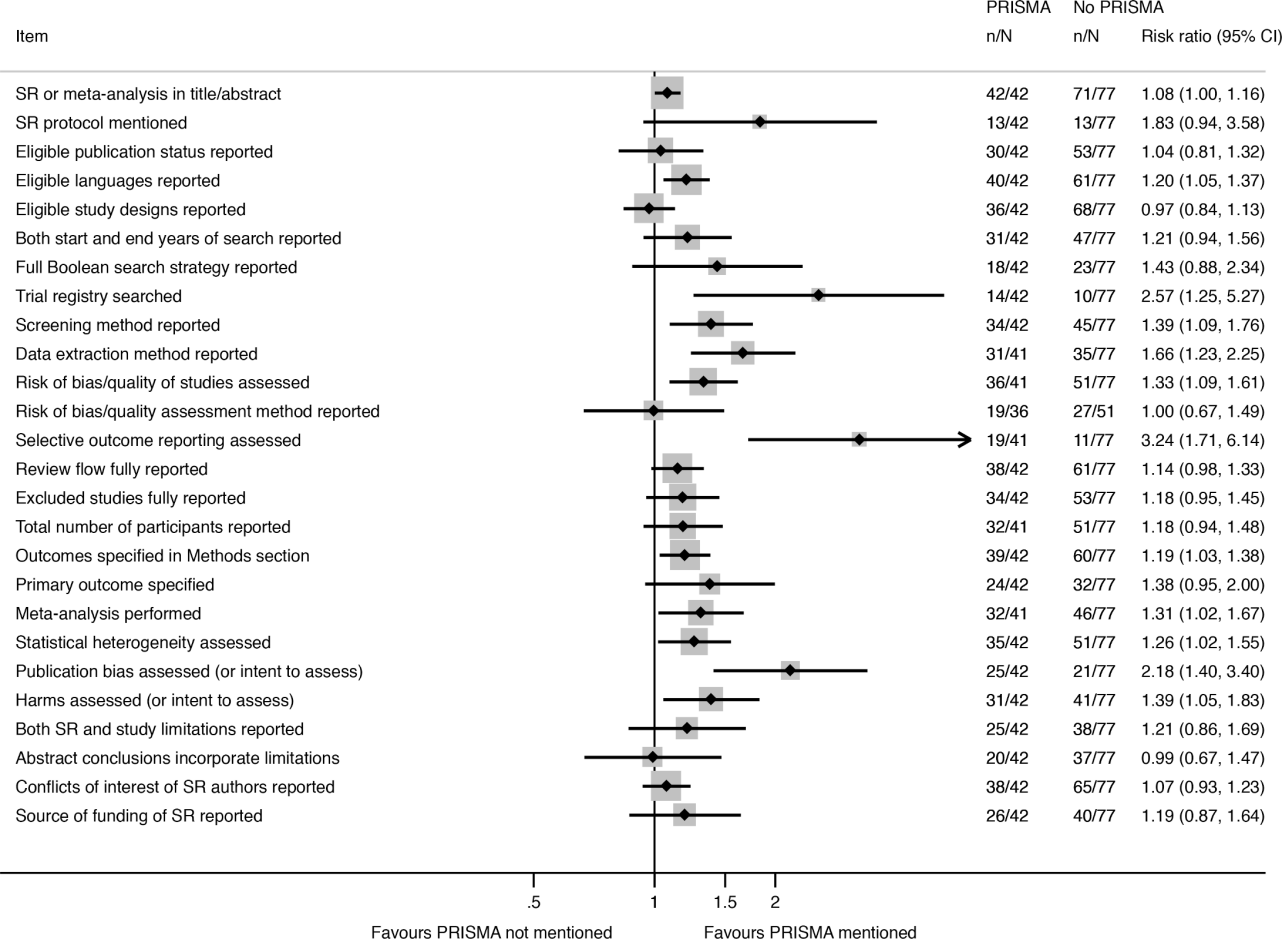
Unadjusted risk ratio associations between reporting characteristics and self-reported use of PRISMA in non-Cochrane therapeutic SRs.

Nearly all of the 26 reporting characteristics of therapeutic SRs that we analyzed according to Cochrane status and self-reported use of the PRISMA Statement were reported more often in SRs that were produced by Cochrane (Fig 2) or that reported that they used the PRISMA Statement (Fig 3). The differences were larger and more often statistically significant in the Cochrane versus non-Cochrane comparison.

### Comparison with 2004 Sample of SRs

The SRs we examined differed in several ways from the November 2004 sample. In 2014, review author teams were larger, and many more SRs were produced by Chinese authors (up from <3% of all SRs in 2004 to 21% in 2014) (Table 4). The proportion of therapeutic SRs decreased (from 71% to 55% of all SRs), coupled with a rise in epidemiological SRs over the decade (from 13% to 25%). Five ICD-10 categories—neoplasms, infections, diseases of the circulatory system, diseases of the digestive system, and mental and behaviour disorders—were the most common conditions in both samples, while two other categories were common in 2014: diseases of the musculoskeletal system and endocrine, nutritional, and metabolic diseases. The proportion of SRs that were Cochrane reviews slightly decreased (from 20% to 15% of all SRs).

**Table 4:** Comparison of the epidemiology of systematic reviews in 2004 and 2014.

Characteristic	Category	Year	
		2004 (*n* = 300)	2014 (*n* = 300)
**Total number of journals**		132	185
**Number of SRs per journal**	1	102/132 (77%)	141/185 (76%)
	2	21/132 (16%)	30/185 (16%)
	3	6/132 (5%)	8/185 (4%)
	≥4	3/132 (2%)	6/185 (3%)
**Journal impact factor**	0.0–5.0	106 (35%)	187 (62%)
	5.1–10	19 (6%)	74 (25%)
	10.1–15	1 (1%)	4 (1%)
	>15	4 (1%)	5 (2%)
	No impact factor	170 (57%)	30 (10%)
**Number of authors**	1	24 (8%)	5 (2%)
	2–3	125 (42%)	64 (21%)
	4–6	128 (43%)	162 (54%)
	≥7	23 (8%)	69 (23%)
**Country of corresponding author**	China	<10 (<3%)	62 (21%)
	UK	76 (25%)	47 (16%)
	US	68 (23%)	46 (15%)
	Canada	28 (9%)	27 (9%)
	The Netherlands	17 (6%)	18 (6%)
	Australia	31 (10%)	16 (5%)
	Germany	10 (3%)	10 (3%)
	Other (<10 reviews/country, 30 countries)	60 (20%)	74 (25%)
**Focus of review**	Therapeutic (treatment/prevention)	213 (71%)	164 (55%)
	Epidemiology (prevalence, associations/etiology)	38 (13%)	74 (25%)
	Diagnosis/prognosis	23 (8%)	33 (11%)
	Other (e.g., education, review of psychometric properties, barriers analysis, cost of illness)	46 (15%)	29 (10%)
**Common ICD-10 codes**	Neoplasms (including cancers, carcinomas, tumors)	22 (7%)	49 (16%)
	Infections and parasitic diseases	20 (7%)	41 (14%)
	Diseases of the circulatory system	33 (11%)	34 (11%)
	Diseases of the digestive system	20 (7%)	25 (8%)
	Endocrine, nutritional, and metabolic diseases	—	23 (8%)
	Mental and behaviour disorders	40 (13%)	22 (7%)
	Diseases of the musculoskeletal system	—	20 (7%)
**Cochrane review**		60 (20%)^a^	45 (15%)

Data given as number (percent).

^a^In 2004, Cochrane reviews were published quarterly, of which 125 were indexed in November 2004. The value presented here has been standardized to a monthly estimate.

There were more updates of previous Cochrane therapeutic SRs in the 2014 sample (38% in 2004 versus 56% in 2014) (Table 5). Also, more SRs included meta-analysis (52% in 2004 versus 63% in 2014). In contrast, there was a reduction in the median number of included studies in SRs in 2014, and in the percentage of therapeutic SRs considering harms (75% in 2004 versus 69% in 2014) or cost (24% versus 13%).

**Table 5:** Comparison of the epidemiological characteristics of systematic reviews in 2004 and 2014, subgrouped by focus of SR.

Characteristic	Percent or Median in 2004 and 2014, and Direction of Change between Time Points					
	All	Therapeutic (Cochrane)	Therapeutic (Non-Cochrane)	Epidemiology	Diagnosis/Prognosis	Other
**Journal type**						
General	9% ↑ 12%	0% = 0%	18% = 18%	11% ↑ 14%	17% ↓ 12%	17% ↓ 7%
Specialty	91% ↓ 88%	100% = 100%	82% = 82%	90% ↓ 86%	83% ↑ 88%	85% ↑ 93%
**Number of authors**	4 ↑ 5	3 ↑ 4	4 ↑ 5	3 ↑ 5	4 ↑ 5	4 = 4
**Update of a previous systematic review**	18% ↓ 10%	38% ↑ 56%	2% ↑ 4%	5% ↓ 0%	0% ↑ 3%	11% ↓ 0%
**Types of interventions**						
Pharmacological	47% ↓ 46%	67% ↓ 51%	53% ↓ 45%	NA	NA	NA
Non-pharmacological	38% ↑ 46%	33% ↑ 38%	49% = 49%	NA	NA	NA
**Number of included studies**	16 ↓ 15	8 ↑ 9	23 ↓ 14	31 ↓ 17	39 ↓ 15	27 ↓ 18
**Number of included participants**	1,112 ↑ 2,072	769 ↑ 1,113	1,137 ↑ 1,565	2,189 ↑ 8,154	14,523 ↓ 1,436	1,644 ↓ 805
**Meta-analysis performed**	54% ↑ 63%	69% ↑ 71%	48% ↑ 66%	37% ↑ 66%	48% ↑ 76%	28% ↓ 17%
**Harms considered** ^**a**^	75% ↓ 69%	86% ↑ 91%	57% ↑ 61%	NA	NA	NA
**Economics (i.e., costs) considered** ^**a**^	24% ↓ 13%	31% ↓ 16%	16% ↓ 7%	NA	NA	NA

^a^“Considered” means that data for the outcome were reported or the authors planned to collect data for the outcome if such data were reported in the included studies.

NA, not applicable.

**Fig 4:**
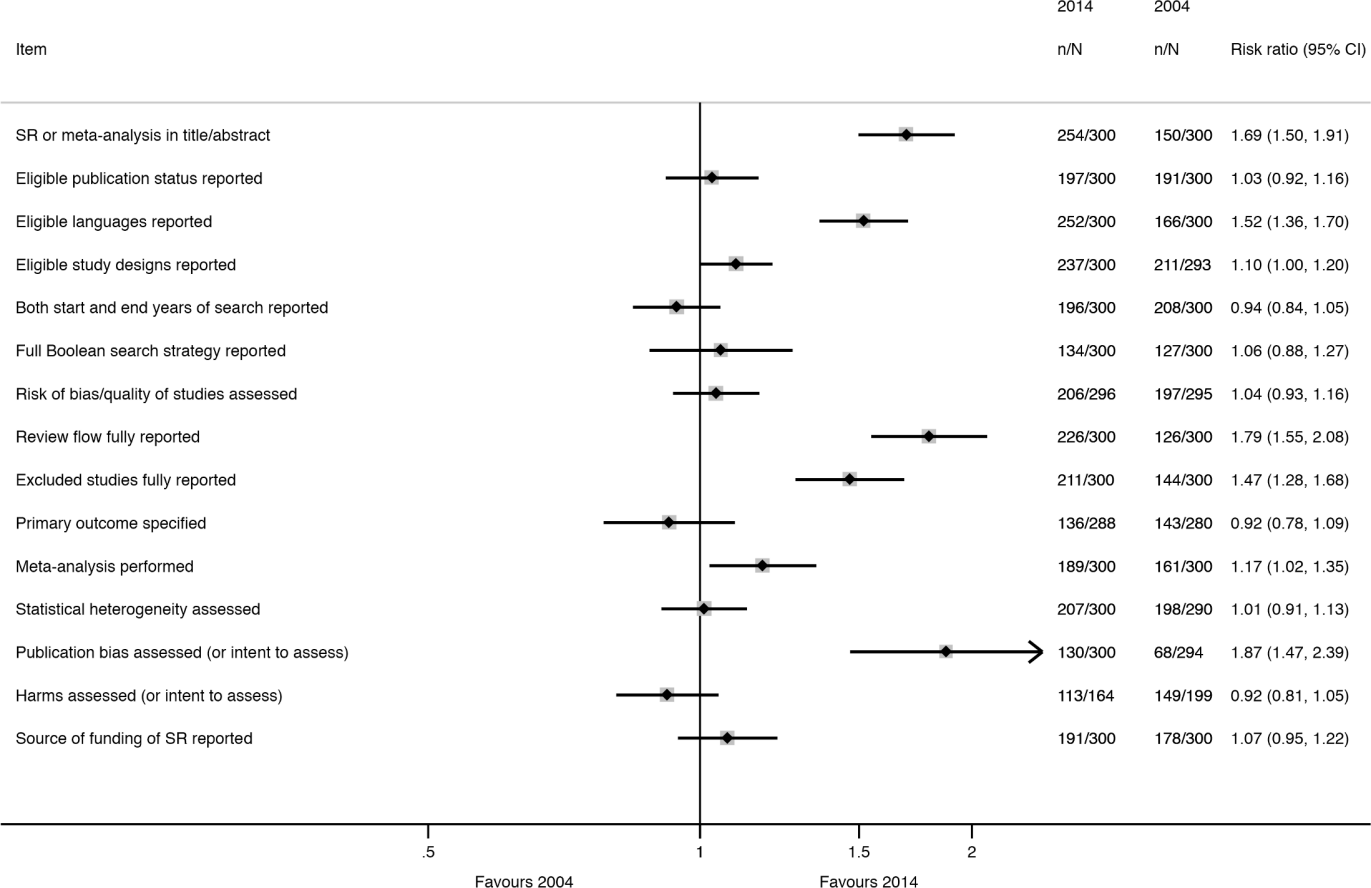
Unadjusted risk ratio associations between reporting characteristics and year: 2004 versus 2014.

Many characteristics were reported more often in 2014 than in 2004 (Fig 4); however, the extent of improvement varied depending on the item and the type of SR (Table 6). The following were reported much more often regardless of the type of SR: eligible language criteria (55% of all SRs in 2004 versus 84% in 2014), review flow (42% versus 78% of all SRs), and reasons for exclusion of full text articles (48% versus 70% of all SRs). Risk of bias/quality assessment was reported more often in 2014 in non-Cochrane therapeutic (49% in 2004 versus 74% in 2014), epidemiology (30% versus 59%), and diagnosis/prognosis (13% versus 67%) SRs but less so in other SRs (42% in 2004 versus 38% in 2014). A primary outcome was specified more often in 2014 in non-Cochrane therapeutic SRs (37% in 2004 versus 48% in 2014) and epidemiology SRs (25% versus 40%) but much less often in other SRs (24% in 2004 versus 3% in 2014). Despite the large improvements for some SR types, many of the 2014 percentages are less than ideal. Further, there was little change or a slight worsening in the reporting of several features, including the mention of a SR protocol in non-Cochrane SRs (14% in 2004 versus 13% in 2014), presentation of a full Boolean search strategy for at least one database (42% versus 45% of all SRs), and reporting of funding source of the SR (59% versus 64% of all SRs).

**Table 6:** Comparison of the reporting characteristics of systematic reviews in 2004 and 2014.

Characteristic	Percent or Median in 2004 and 2014, and Direction of Change between Time Points					
	All	Therapeutic (Cochrane)	Therapeutic (Non-Cochrane)	Epidemiology	Diagnosis/Prognosis	Other
**“Systematic review” or “meta-analysis” used in the title or abstract**	50% ↑ 85%	39% ↓ 33%	68% ↑ 95%	50% ↑ 93%	65% ↑ 97%	35% ↑ 86%
**SR protocol mentioned**	46% ↓ 26%	98% = 98%	11% ↑ 22%	8% ↓ 4%	4% ↑ 6%	22% ↓ 7%
**Eligible publication status**						
Both published and unpublished studies	41% ↓ 39%	69% ↑ 91%	22% ↑ 41%	18% = 18%	22% ↓ 18%	30% ↓ 24%
Only published studies	23% ↑ 27%	6% ↓ 4%	33% ↓ 28%	47% ↓ 34%	26% ↑ 27%	28% ↑ 38%
Not reported	36% ↓ 34%	26% ↓ 4%	45% ↓ 30%	34% ↑ 49%	52% ↑ 55%	41% ↓ 38%
**Eligible languages**						
All languages considered	37% ↑ 43%	62% ↑ 82%	26% ↑ 40%	13% ↑ 39%	17% ↑ 30%	15% ↑ 17%
English only	16% ↑ 31%	1% ↑ 2%	31% ↑ 37%	26% ↑ 30%	30% ↑ 36%	26% ↑ 45%
Mixed (English and a specific language other than English)	2% ↑ 10%	0% ↑ 2%	3% ↑ 8%	5% ↑ 14%	9% ↑ 15%	4% ↑ 21%
Not reported	45% ↓ 16%	37% ↓ 13%	40% ↓ 15%	55% ↓ 18%	44% ↓ 18%	54% ↓ 17%
**Eligibility criteria based on study designs reported**	72% ↑ 79%	100% = 100%	63% ↑ 87%	39% ↑ 76%	27% ↑ 52%	55% ↓ 52%
**Number of databases searched**	3 ↑ 4	4 ↑ 5	2 ↑ 3	2 ↑ 3	2 ↑ 3	3 ↑ 5
**Years of coverage reported**						
Both start and end dates are reported for all databases	69% ↓ 65%	83% ↑ 91%	58% ↑ 66%	55% ↓ 53%	70% ↓ 58%	63% ↑ 66%
Partially—start and end dates are reported for only one of many databases, or only the end date is reported for all databases	16% ↑ 29%	12% ↓ 9%	21% ↑ 29%	13% ↑ 41%	9% ↑ 39%	20% ↑ 21%
**Search terms reported**						
Full Boolean search logic reported for one or more databases	42% ↑ 45%	78% ↑ 98%	18% ↑ 34%	11% ↑ 35%	17% ↑ 39%	28% ↑ 34%
Main index terms (e.g., MeSH) reported	17% ↓ 12%	11% ↓ 0%	22% ↓ 14%	18% ↓ 14%	17% ↓ 15%	22% ↓ 10%
Free text words reported	26% ↑ 46%	9% ↓ 0%	35% ↑ 54%	40% ↑ 55%	48% ↑ 54%	37% ↑ 48%
No search terms reported	12% ↓ 5%	1% ↓ 0%	22% ↓ 1%	26% ↓ 3%	13% ↓ 3%	11% ↓ 7%
**Study risk of bias/quality formally assessed**	67% ↑ 70%	100% = 100%	49% ↑ 74%	30% ↑ 59%	13% ↑ 67%	42% ↓ 38%
**Review flow reported**						
Completely in PRISMA-like flow diagram	7% ↑ 69%	4% ↑ 51%	11% ↑ 79%	8% ↑ 62%	4% ↑ 79%	7% ↑ 59%
Completely in text/table only	35% ↓ 7%	38% ↓ 11%	35% ↓ 4%	24% ↓ 8%	39% ↓ 6%	44% ↓ 7%
Partially reported	33% ↓ 13%	48% ↓ 16%	19% ↓ 12%	24% ↓ 15%	22% ↓ 6%	22% ↓ 14%
Not reported	31% ↓ 12%	14% ↑ 22%	43% ↓ 5%	53% ↓ 15%	39% ↓ 9%	35% ↓ 21%
**Reasons for exclusion of full text articles reported**						
Reasons for all excluded articles reported in text/table or PRISMA-like flow diagram	48% ↑ 70%	60% ↑ 87%	45% ↑ 69%	23% ↑ 54%	43% ↑ 76%	46% ↓ 45%
Partially—reasons for only some excluded articles reported	40% ↓ 9%	39% ↓ 9%	38% ↓ 8%	50% ↓ 18%	30% ↓ 3%	35% ↓ 3%
Not reported for any articles	17% ↑ 20%	2% ↓ 0%	25% ↓ 19%	32% ↓ 26%	30% ↓ 12%	26% ↑ 52%
**Primary outcome specified**	51% ↓ 47%	77% ↑ 96%	37% ↑ 48%	25% ↑ 40%	15% ↑ 18%	24% ↓ 3%
**Statistical heterogeneity investigated**						
Using statistical methods or qualitatively assessed (e.g., via narrative discussion)	68% ↑ 69%	93% ↓ 73%	54% ↑ 72%	47% ↑ 70%	45% ↑ 85%	46% ↓ 28%
Using statistical methods when meta-analysis performed	91% ↑ 93%	100% = 100%	83% ↑ 91%	79% ↑ 92%	73% ↑ 96%	92% ↓ 60%
**Risk of publication bias assessed (or intent to assess)**	23% ↑ 43%	32% ↑ 78%	18% ↑ 39%	18% ↑ 45%	9% ↑ 45%	12% ↓ 3%
**Possibility of publication bias discussed/considered in results, discussion, or conclusion**	31% ↑ 47%	39% ↑ 64%	31% ↑ 46%	29% ↑ 50%	18% ↑ 55%	14% ↓ 7%
**Source of funding of the SR**						
Non-profit	48% ↓ 47%	65% ↑ 84%	36% ↑ 40%	26% ↑ 41%	26% ↑ 27%	41% ↑ 59%
For-profit	2% ↑ 3%	1% ↓ 0%	5% ↓ 3%	3% ↓ 3%	0% = 0%	2% ↑ 10%
Mixed	6% ↓ 1%	7% ↓ 0%	6% ↓ 1%	5% ↓ 1%	9% ↓ 0%	4% ↓ 0%
Authors specified there was no funding	1% ↑ 13%	0% ↑ 11%	2% ↑ 12%	0% ↑ 15%	0% ↑ 21%	2% ↑ 7%
Not reported	41% ↓ 36%	19% ↓ 4%	50% ↓ 45%	66% ↓ 41%	65% ↓ 51%	50% ↓ 24%

## Discussion

We estimate that more than 8,000 SRs are being indexed in MEDLINE annually, corresponding to a 3-fold increase over the last decade. The majority of SRs indexed in February 2014 addressed a therapeutic question and were conducted by authors based in China, the UK, or the US; they included a median of 15 studies involving 2,072 participants. Meta-analysis was performed in 63% of SRs, mostly using standard pairwise methods. Study risk of bias/quality assessment was performed in 70% of SRs, but rarely incorporated into the analysis (16%). Few SRs (7%) searched sources of unpublished data, and the risk of publication bias was considered in less than half of SRs. Reporting quality was highly variable; at least a third of SRs did not mention using a SR protocol or did not report eligibility criteria relating to publication status, years of coverage of the search, a full Boolean search logic for at least one database, methods for data extraction, methods for study risk of bias assessment, a primary outcome, an abstract conclusion that incorporated study limitations, or the funding source of the SR. Cochrane SRs, which accounted for 15% of the sample, had more complete reporting than all other types of SRs. Reporting has generally improved since 2004, but remains suboptimal for many characteristics.

### Explanation of Results and Implications

The increase in SR production from 2004 to 2014 may be explained by several positive changes over the decade. The scientific community and health care practitioners may have increasingly recognized that the deluge of published research over the decade requires integration, and that a synthesis of the literature is more reliable than relying on the results of single studies. Some funding agencies (e.g., the UK National Institute for Health Research and the Canadian Institutes of Health Research) now require applicants to justify their applications for research funding with reference to a SR, which the applicants themselves must perform if one does not exist [26]. Further, some countries, particularly China, have developed a research culture that places a strong emphasis on the production of SRs [27]. Also, the development of free software to perform meta-analyses (e.g., RevMan [28], MetaXL [29], R [30,31]) has likely contributed to its increased use.

There are also some unsavory reasons for the proliferation of SRs. In recent years, some countries have initiated financial incentives to increase publication rates (e.g., more funding for institutions that publish more articles or cash bonuses to individuals per article published) [32]. Further, appointment and promotion committees often place great emphasis on the number of publications an investigator has, rather than on the rigor, transparency, and reproducibility of the research [4]. Coupled with the growing recognition of the value of SRs, investigators may be strongly motivated to publish a large number of SRs, regardless of whether they have the necessary skills to perform them well. In addition, the proliferation of new journals over the decade has made it more likely that authors can successfully submit a SR for publication regardless of whether one on the same topic has been published elsewhere. This has resulted in a large number of overlapping SRs (one estimate suggests 67% of meta-analyses have at least one overlapping meta-analysis within a 3-y period) [33]. Such overlap of SR questions is not possible in the *Cochrane Database of Systematic Reviews*, which may explain why the proportion of Cochrane SRs within the broader SR landscape has diminished.

The conduct of SRs was good in some respects, but not others. Examples of good conduct are that nearly all SRs searched more than one bibliographic database, and the majority performed dual-author screening, data extraction, and risk of bias assessment. However, few SRs searched sources of unpublished data (e.g., trial registries, regulatory databases), despite their ability to reduce the impact of reporting biases [34,35]. Also, an appreciable proportion of SRs (particularly epidemiology and diagnosis/prognosis SRs) did not assess the risk of bias/quality of the included studies. In addition, the choice of meta-analysis model in many SRs was guided by heterogeneity statistics (e.g., *I* ^2^), a practice strongly discouraged by leading SR organizations because of the low reliability of these statistics [19,20]. It is therefore possible that some systematic reviewers inappropriately generated summary estimates, by ignoring clinical heterogeneity when statistical heterogeneity was perceived to be low. Further, a suboptimal number of therapeutic SRs considered the harms of interventions. It is possible that review authors did not comment on harms when none were identified in the included studies. However, reporting of both zero and non-zero harm events is necessary so that patients and clinicians can determine the risk–benefit profile of an intervention [36]. To reduce the avoidable waste associated with these examples of poor conduct of SRs, strategies such as formal training of biomedical researchers in research design and analysis and the involvement of statisticians and methodologists in SRs are warranted [4].

Cochrane SRs continue to differ from their non-Cochrane counterparts. Completeness of reporting is superior in Cochrane SRs, possibly due to the use of strategies in the editorial process that promote good reporting (such as use of the Methodological Expectations of Cochrane Intervention Reviews [MECIR] standards [37]). Also, word limits or unavailability of online appendices in some non-Cochrane journals may lead to less detailed reporting. Cochrane SRs tend to include fewer studies, which may be partly due to the reviews more often restricting inclusion to randomized trials only. However, fewer studies being included could also result from having a narrower review question (in terms of the patients, interventions, and outcomes that are addressed). Further research should explore the extent to which Cochrane and non-Cochrane SRs differ in scope, and hence applicability to clinical practice.

It is notable that reporting of only a few characteristics improved substantially over the decade. For example, the SRs in the 2014 sample were much more likely to present a review flow and reasons for excluded studies than SRs in the 2004 sample. This was most often done using a PRISMA flow diagram [7], suggesting that this component of the PRISMA Statement has been successfully adopted by the SR community. However, 2014 SRs were slightly less likely than their 2004 counterparts to identify an outcome as “primary” and to report both the start and end years of the search, and the number of SRs reporting the source of funding increased only marginally. We do not believe that the smaller changes in reporting of some characteristics is due to their receiving less emphasis in the original paper by Moher et al. [6] or the PRISMA Statement [7], because neither emphasized any characteristic over others. Therefore, more research is needed to determine which characteristics authors think are less important to report in a SR, and why.

Mention of the PRISMA Statement [7], perhaps a surrogate for actual use, appears to be associated with more complete reporting. However, reporting of many SRs remains poor despite the availability of the PRISMA Statement since 2009. There are several possible reasons for this. Some authors may still be unaware of PRISMA or assume that they already know how to report a SR completely. The extent to which journals endorse PRISMA is highly variable, with some explicitly requiring authors to submit a completed checklist at the time of manuscript submission, others only recommending its use in the instructions to authors, and many not referring to it at all [38,39]. Also, some PRISMA items include multiple elements (e.g., item 7 asks authors to describe the databases searched, whether authors were contacted to identify additional trials, the years of coverage of the databases searched, and the date of the last search). Some authors may assume that they have adequately addressed an item if they report at least one element. Also, authors may consider PRISMA only after spending hours drafting and refining their manuscript with co-authors, a point when they may be less likely to make the required changes [40].

Our findings suggest that strategies other than the passive dissemination of reporting guidelines are needed to address the poor reporting of SRs. One strategy is to develop software that facilitates the completeness of SR reporting [41]. For example, Barnes and colleagues recently developed an online writing tool based on the CONSORT Statement [40]. The tool is meant to be used by authors when writing the first draft of a randomized trial report and consists of bullet points detailing all the key elements of the corresponding CONSORT item(s) to be reported, with examples of good practice. Medical students randomly assigned to use the tool over a 4-h period reported trial methods more completely [40]; thus, a similar tool based on the PRISMA Statement is worth exploring. Also, journal editors could receive certified training in how to endorse and implement PRISMA and facilitate its use by peer reviewers [42]. Further, collaboration between key stakeholders (funders, journals, academic institutions) is needed to address poor reporting [26].

More research is needed on the risk of bias in SRs that is associated with particular methods. We observed that a considerable proportion of therapeutic SRs (40%) had potentially misleading conclusions because the limitations of the evidence on which the conclusions were based were not taken into consideration. This is a problem because some users of SRs only have access to the abstract and may be influenced by the misleading conclusions to implement interventions that are either ineffective or harmful [9]. We have not explored in this study the extent to which the results of the SRs we examined were biased. Such bias can occur for several reasons, including use of inappropriate eligibility criteria, failure to use methods that minimize error in data collection, selective inclusion of the most favourable results from study reports, inability to access unpublished studies, and inappropriate synthesis of clinically heterogeneous studies [43]. Determining how often the results of SRs are biased is important because major users of SRs such as clinical practice guideline developers tend to rely on the results (e.g., intervention effect estimates) rather than conclusions when formulating recommendations [44]. We only recorded whether methodological characteristics were reported or not, rather than evaluating how optimal each method was. Further, exploring whether non-reporting of a method is associated with biased results is problematic, because non-compliance with reporting guidelines is not necessarily an indicator of a SR’s methodological quality. That is, some review authors may use optimal methods but fail to clearly specify those methods for non-bias-related reasons (e.g., word limits). In future, investigators could apply to the SRs in our sample a tool such as the ROBIS tool [45], which guides appraisers to make judgements about the risk of bias at the SR level (rather than the study level) due to several aspects of SR conduct and reporting.

### Strengths and Limitations

There are several strengths of our methods. We used a validated search filter to identify SRs, and screened each full text article twice to confirm that it met the eligibility criteria. Screening each article provides a more reliable estimate of SR prevalence than relying on the search filters for SRs, which we found retrieved many non-systematic reviews and other knowledge syntheses. We did not restrict inclusion based on the focus of the SR and, thus, unlike previous studies [14,15], were able to collect data on a broader cross-section of SRs.

There are also some limitations to our study. Our results reflect what was reported in the articles, and it is possible that some SRs were conducted more rigorously than was specified in the report, and vice versa. Our findings may not generalize to SRs indexed outside of MEDLINE or published in a language other than English. Two authors independently and in duplicate extracted data on only a 10% random sample of SRs. We attempted to minimize data extraction errors by independently verifying data for 42/88 “problematic items” (i.e., those where there was at least one discrepancy between two authors in the 10% random sample). We cannot exclude the possibility of errors in the non-verified data items, although we consider the risk to be low given that the error rate for these items was 0% in the random sample. Also, our results concerning some types of SRs (e.g., diagnosis/prognosis, other) were based on small samples, so should be interpreted with caution. Further, searching for articles indexed in MEDLINE, rather than published, during the specified time frame means that we examined a small number of SRs (8/300 [3%]) with more than a year’s delay in indexing after publication. However, inclusion of these few articles is unlikely to have affected our findings.

Some terminology contained within the PRISMA-P definition of a SR may be interpreted differently by different readers (e.g., “systematic search” and “explicit, reproducible methodology”). Hence, it is possible that others applying the PRISMA-P definition may have reached a slightly different estimate of SR prevalence than we did. We tried to address this by also reporting a SR prevalence that included articles consistent with the less explicit definition used by Moher et al. [6]. Also, any observed improvements in reporting since 2004 may partly be attributed to our use of a more stringent definition of SRs in 2014, which required articles to meet more minimum reporting requirements. Hence, we may have slightly overestimated the improvements in reporting from 2004 to 2014 and underestimated the true scale of poor reporting in SRs.

### Conclusion

An increasing number of SRs are being published, and many are poorly conducted and reported. This is wasteful for several reasons. Poor conduct can lead to SRs with misleading results, while poor reporting prevents users from being able to determine the validity of the methods used. Strategies are needed to increase the value of SRs to patients, health care practitioners, and policy makers.

## Supporting Information

S1 Protocol Study protocol. (DOCX)

S1 Forms Screening and data extraction forms. (DOCX)

S1 Results Supplementary results. (DOCX)

## Abbreviations

IQR: interquartile range
SR: systematic review
