# Supplementary material for: Epidemiology and Reporting Characteristics of Systematic Reviews of Biomedical Research: A Cross-Sectional Study
Source: PLoS Med. 2016 May 24;13(5):e1002028. doi: 10.1371/journal.pmed.1002028 (PMC4878797; doi:10.1371/journal.pmed.1002028)
Supplement: S1 Protocol — (DOCX) [file pmed.1002028.s001.docx]

**Study protocol: Epidemiology and reporting characteristics of systematic reviews (2014 update)**

Matthew J Page, Larissa Shamseer, Douglas G Altman, Jennifer Tetzlaff, Margaret Sampson, Andrea C Tricco, Ferrán Catalá-López, Lun Li, Emma Reid, Rafael Sarkis-Onofre, David Moher

28 February 2014

**Background**

Moher et al. previously described the epidemiological, descriptive and reporting characteristics of a representative sample of systematic review (SR) reports indexed in MEDLINE® in November 2004 (Moher 2007). Ten years have elapsed since 2004 and the systematic review landscape has changed considerably, with the publication of a reporting guideline for SRs (Moher 2009), the launch of the Institute of Medicine’s standards for SRs of comparative effectiveness research (IOM 2011), methodological developments such as a new tool to assess the risk of bias of included studies in SRs (Higgins 2011), and the proliferation of new open-access journals, in particular *Systematic Reviews*, a journal specifically for SRs and associated research (Moher 2012). While recent studies have evaluated either the prevalence of SRs (e.g. Bastian 2010) or reporting characteristics of SRs of particular clinical areas (e.g. Gianola 2013, Turner 2013), a direct replication of the Moher 2007 study in a more recent sample of SRs has not been conducted. Therefore, we considered it timely to update the Moher 2007 study.

**Objectives**

To investigate the epidemiological, descriptive and reporting characteristics of SRs indexed in MEDLINE® in February 2014.

**Methods**

***Eligibility criteria***

To be eligible for inclusion, articles will have to meet the following definition of a SR, as used in Moher 2007: *There is no standard definition of an SR. We counted a report as an SR if the authors’ stated objective was to summarize evidence from multiple studies and the article described explicit methods, regardless of the details provided*. We will only include published SRs written in English.

***Searching***

To provide a reliable summary of the literature, we will search for SRs indexed throughout one calendar month. We will select February 2014 as it is the month closest to when the protocol for this study was drafted. We will search MEDLINE® using the search strategy reported in Moher 2007 (see Appendix 1). The search results will be uploaded into online review software, DistillerSR®.

***Screening***

Screening will be undertaken using DistillerSR®. A form for screening of titles and abstracts will be pilot-tested on five records. Subsequently, all titles and abstracts will be screened using liberal acceleration (where two reviewers need to independently exclude a record while only one reviewer needs to include a record). We will retrieve the full text of any citations meeting our eligibility criteria or for which eligibility remains unclear. A form for screening full text articles will be pilot-tested on five articles. Subsequently, pairs of reviewers (*number to be determined*) will independently screen all full text articles. Any discrepancies in screening of titles and abstracts and full text articles will be resolved via discussion or adjudication by a third reviewer if necessary.

***Data extraction***

If more than 300 SRs are identified in the search, we will perform data extraction on a stratified random sample of 300 SRs (stratification by Cochrane versus non-Cochrane SR). Data will be collected using a standardized data extraction form containing (*number to be determined*) items. All data extractors will independently pilot-test the form on five included SRs to ensure consistency in interpretation of data items. Subsequently, data from each SR will be independently extracted by one of several reviewers, with a 10% random sample extracted in duplicate. Any discrepancies in the data extracted will be resolved via discussion or adjudication by a third reviewer if necessary. The data extraction items will be based on those reported in Moher 2007 and Turner 2013. Any other items nominated and agreed upon by the review team will also be included.

***Data analysis***

All analyses will be performed using Stata version 11 software. The analysis will be descriptive, with data summarized as frequency for categorical items or median and interquartile range for continuous items. We will also compare the epidemiological, descriptive and reporting characteristics of SRs in the 2014 sample to SRs in the 2004 sample, calculating risk ratios for key characteristics.

***Subgroup Analyses***

We will undertake the following subgroup analyses to compare reviews in terms of their reporting characteristics:

- Cochrane versus non-Cochrane SR;
- SRs with versus without a protocol;
- SRs registered versus not registered.

***Sensitivity Analyses***

We will undertake sensitivity analyses to investigate whether the estimate of SR prevalence is influenced by use of different definitions of SRs. Specifically, we will estimate the prevalence of SRs defined according to the PRISMA-P 2015 definition.

**Note: the protocol was revised in August 2014, in that articles had to meet the PRISMA-P 2015 definition to be included in the study, and a sensitivity analysis was performed by counting articles that met the Moher 2007 definition. We revised the initial plan because use of the Moher 2007 definition ignores the evolution of SR terminology over the decade.**

**References**

Bastian H, Glasziou P, Chalmers I. Seventy-five trials and eleven systematic reviews a day: how will we ever keep up? PLoS Medicine 2010;7(9):e1000326.

Gianola S, Gasparini M, Agostini M, Castellini G, Corbetta D, Gozzer P, et al. Survey of the reporting characteristics of systematic reviews in rehabilitation. Physical Therapy 2013;93(11)1456-66.

Higgins JPT, Altman DG, Gøtzsche PC, Jüni P, Moher D, Oxman AD, et al. The Cochrane Collaboration’s tool for assessing risk of bias in randomised trials. BMJ 2011;343:d5928.

IOM (Institute of Medicine). 2011. Finding What Works in Health Care: Standards for Systematic Reviews. Washington, DC: The National Academies Press.

Kastner M, Tricco AC, Soobiah C, Lillie E, Perrier L, Horsley T, et al. What is the most appropriate knowledge synthesis method to conduct a review? Protocol for a scoping review. BMC Medical Research Methodology 2012;12:114.

Moher D, Tetzlaff J, Tricco AC, Sampson M, Altman DG. Epidemiology and reporting characteristics of systematic reviews. PLoS Medicine 2007;4(3):e78.

Moher D, Liberati A, Tetzlaff J, Altman DG, The PRISMA Group. Preferred Reporting Items for Systematic Reviews and Meta- Analyses: The PRISMA Statement. PLoS Medicine 2009;6(7):e1000097.

Moher D, Stewart L, Shekelle P. Establishing a new journal for systematic review products. Systematic Reviews 2012;1:1.

Montori VM, Wilczynski NL, Morgan D, Haynes RB. Optimal search strategies for retrieving systematic reviews from Medline analytical survey. BMJ 2005;330:68.

Turner L, Galipeau J, Garritty C, Manheimer E, Wieland LS, Yazdi F, et al. An evaluation of epidemiological and reporting characteristics of complementary and alternative medicine (CAM) systematic reviews (SRs). PLoS ONE 2013;8(1):e53536.

**Appendix 1: Search strategy**

1. 201402$.ed
2. limit 1 to English
3. 2 and (cochrane database of systematic reviews.jn. or search.tw. or metaanalysis.pt. or medline.tw. or systematic review.tw. or ((metaanalysis.mp,pt. or review.pt. or search$.tw.) and methods.ab.))
