# Supplementary material for: Epidemiology and Reporting Characteristics of Systematic Reviews of Biomedical Research: A Cross-Sectional Study
Source: PLoS Med. 2016 May 24;13(5):e1002028. doi: 10.1371/journal.pmed.1002028 (PMC4878797; doi:10.1371/journal.pmed.1002028)
Supplement: S1 Forms — (DOCX) [file pmed.1002028.s002.docx]

**Eligibility criteria screening and data extraction forms**

**Title/abstract screening form**

| **Question** | **Response options** |
| --- | --- |
| 1. Does the report present a knowledge synthesis? A knowledge synthesis summarizes all pertinent studies on a specific question, can improve the understanding of inconsistencies in diverse evidence, and can define future research agendas (Kastner et al. BMC Medical Research Methodology 2012;12:114) | Yes |
|  | Unclear |
|  | No |
| 1. Was it the authors’ stated objective to summarize evidence from multiple studies, and did the article describe explicit methods (regardless of the details provided)? Note that SRs are eligible regardless of the focus of the review, i.e. treatment, prevention, diagnostic, prognostic, epidemiology (e.g. prevalence), other (e.g. education, cost analysis). | Yes |
|  | Unclear |
|  | No |
| 1. Does the report represent a completed, original study (i.e. NOT a protocol or a summary of published findings)? | Yes |
|  | Unclear |
|  | No |

**Full text screening form (preliminary)**

| **Question** | **Response options** |
| --- | --- |
| 1. Does the report present a knowledge synthesis? A knowledge synthesis summarizes all pertinent studies on a specific question, can improve the understanding of inconsistencies in diverse evidence, and can define future research agendas (Kastner et al. BMC Medical Research Methodology 2012;12:114) | Yes |
|  | Unclear |
|  | No |
| 1. Does the report represent a completed, original synthesis (i.e. NOT a protocol or a summary of published findings)? | Yes |
|  | Unclear |
|  | No |
| 1. What type of knowledge synthesis is presented in the report? | Systematic review: the authors stated objective was to summarise evidence from multiple studies and the article described explicit methods, regardless of the details provided (Moher et al. PLoS Medicine 2007;4:e78) |
|  | Narrative review: an informal approach used to describe the selection, chronicling, and ordering of primary evidence (or no approach described) to produce an account of the evidence with commentary and interpretation. It can ‘integrate’ qualitative and quantitative evidence through narrative juxtaposition (discussing diverse forms of evidence side by side). It is less concerned with assessing evidence quality and more focused on gathering relevant information that provides both context and substance to the authors’ overall argument (Kastner et al. BMC Medical Research Methodology 2012;12:114) |
|  | Non-systematic review with meta-analysis or network meta-analysis: the authors report conducting a meta-analysis or network meta-analysis but did not use systematic review methods to identify and select studies |
|  | Non-systematic review with meta-synthesis: the authors report conducting a meta-synthesis but did not use systematic review methods to identify and select studies. “Meta-synthesis” is an umbrella term used to describe meta-ethnography, meta-summary, meta-study, pragmatic utility, and other methods. These synthesis methods were developed in response to concerns about the relevance and utility of qualitative research, and involve combining separate elements to form a coherent whole using a process of logical deduction. Its aims are to portray an accurate interpretation of a phenomenon and to compare and contrast the constructs of individual studies to reach consensus on a new construction of that phenomenon. It involves: identifying findings, grouping findings into categories and grouping categories into synthesized findings. Meta-synthesis is an integrative and expansionistic method that compares and analyzes in a constructivist way, allowing interpretive themes or key metaphors to emerge from the synthesis (Tricco et al. Unpublished Aug 2014) |
|  | Rapid review: a type of literature review produced using accelerated and/or abbreviated systematic review methods (OHRI Knowledge Synthesis Group meeting on rapid reviews at 2013 Cochrane Colloquium) |
|  | Overview of reviews or umbrella review: a review that uses systematic and explicit methods to summarise evidence from existing systematic reviews (Thomson et al. Research Synthesis Methods 2010;1:198-211) |
|  | Scoping review: form of knowledge synthesis that addresses an exploratory research question aimed at mapping key concepts, types of evidence and gaps in research related to a defined area or field by systematically searching, selecting, and synthesizing existing knowledge (Colquhoun et al. Journal of Clinical Epidemiology 2014) |
|  | Other (please indicate type of knowledge synthesis): |
|  | Unclear/unsure |
| 1. Is the report written in English, French, Spanish, Chinese or Portuguese? | Yes |
|  | No |

**Full text screening form (final)**

| **Question** | **Response options** |
| --- | --- |
| 1. Are methods to identify studies (i.e. a search strategy) explicitly stated? | Yes |
|  | No |
|  | Unsure |
| 1. Are methods of study selection (e.g. eligibility criteria and selection process) explicitly stated? | Yes |
|  | No |
|  | Unsure |
| 1. Are methods of synthesis (or other type of summary) explicitly described? | Yes |
|  | No |
|  | Unsure |
| 1. Do you think that the article in question represents another type of knowledge synthesis, other than a systematic review? | Yes |
|  | No |
|  | Unsure |
| 1. Indicate which of the following review types are applicable: | Narrative review: an informal approach used to describe the selection, chronicling, and ordering of primary evidence to produce an account of the evidence with commentary and interpretation. It can ‘integrate’ qualitative and quantitative evidence through narrative juxtaposition (discussing diverse forms of evidence side by side). It is less concerned with assessing evidence quality and more focused on gathering relevant information that provides both context and substance to the authors’ overall argument (Kastner et al. BMC Medical Research Methodology 2012;12:114) |
|  | Non-systematic review with meta-analysis or network meta-analysis: the authors report conducting a meta-analysis or network meta-analysis but did not use systematic review methods to identify and select studies |
|  | Non-systematic review with meta-synthesis: the authors report conducting a meta-synthesis but did not use systematic review methods to identify and select studies. “Meta-synthesis” is an umbrella term used to describe meta-ethnography, meta-summary, meta-study, pragmatic utility, and other methods. These synthesis methods were developed in response to concerns about the relevance and utility of qualitative research, and involve combining separate elements to form a coherent whole using a process of logical deduction. Its aims are to portray an accurate interpretation of a phenomenon and to compare and contrast the constructs of individual studies to reach consensus on a new construction of that phenomenon. It involves: identifying findings, grouping findings into categories and grouping categories into synthesized findings. Meta-synthesis is an integrative and expansionistic method that compares and analyzes in a constructivist way, allowing interpretive themes or key metaphors to emerge from the synthesis (Tricco et al. Unpublished Aug 2014) |
|  | Rapid review: a type of literature review produced using accelerated or abbreviated systematic review methods (OHRI Knowledge Synthesis Group meeting on rapid reviews at 2013 Cochrane Colloquium) |
|  | Overview of reviews or umbrella review: a review that uses systematic and explicit methods to summarise evidence from existing systematic reviews (Thomson et al. Research Synthesis Methods 2010;1:198-211) |
|  | Scoping review: a form of knowledge synthesis that addresses an exploratory research question aimed at mapping key concepts, types of evidence and gaps in research related to a defined area or field by systematically searching, selecting, and synthesizing existing knowledge (Colquhoun et al. Journal of Clinical Epidemiology 2014) |
|  | Other (please indicate type of knowledge synthesis): |

**Data extraction form**

Data in each SR was collected using the following form by one of five authors. A 10% random sample of SRs was extracted in duplicate, which revealed several items that were discrepant between two reviewers on at least one occasion. Data for these items (indicated by an asterisk * below) were verified for all SRs by one author.

| **Question** | **Response option** |
| --- | --- |
| RefID |  |
| Your initials |  |
| Which journal is the SR published in? |  |
| Is the journal a general or specialty journal? "General" journals are those that cover a range of clinical conditions and intervention types (e.g. NEJM, BMJ, Lancet) whereas "Specialty" journals focus on a specific clinical condition or intervention type (e.g. Diabetes Care, Physical Therapy) | General |
|  | Specialty |
| Number of authors of this SR |  |
| What is the country of the corresponding author? |  |
| Which of the following terms are in the title or abstract of the review? Please choose ONE only | Systematic review |
|  | Meta-analysis |
|  | Both "systematic review" and "meta-analysis" |
|  | Neither |
| Is this a Cochrane review? | Yes |
|  | No |
| Do the authors refer to this an original systematic review or an update of a previous systematic review? | Original systematic review |
|  | Update of a previous systematic review |
| What is the broad ICD-10 category investigated in this systematic review? For guidance, see http://apps.who.int/classifications/icd10/browse/2015/en. Please choose the most relevant disease according to the ICD-10 system (e.g. if the condition is lung cancer, please select "Neoplasms" rather than "Diseases of the respiratory system"). Please choose ONE only | Infections and parasitic diseases |
|  | Neoplasms (incl. cancers, carcinomas, tumors) |
|  | Diseases of the blood and blood forming organs, immune mechanism |
|  | Endocrine, nutritional, and metabolic disease |
|  | Mental and behaviour disorders |
|  | Diseases of the nervous system |
|  | Diseases of the eye and adnexa |
|  | Diseases of the ear and mastoid process |
|  | Diseases of the circulatory system |
|  | Diseases of the respiratory system |
|  | Diseases of the digestive system |
|  | Diseases of the skin and subcutaneous tissue |
|  | Diseases of the musculoskeletal system and connective tissue |
|  | Diseases of the genitourinary system |
|  | Pregnancy, childbirth, and the puerperium |
|  | Certain conditions originating in the perinatal period |
|  | Congenital malformations, deformations, and chromosomal abnormalities |
|  | Symptoms, signs and abnormal clinical and laboratory findings, not elsewhere classified |
|  | Injury, poisoning, and certain other consequences of external causes |
|  | External causes of morbidity and mortality |
|  | Factors influencing health status and contact with health services |
|  | Unclear (please specify) |
|  | Not applicable (please explain) |
| *What was the primary focus of the review? Please choose ONE only | Treatment/Therapeutic |
|  | Prevention |
|  | Diagnosis |
|  | Prognosis |
|  | Epidemiology (e.g. association between exposure and prevalence/incidence) |
|  | Unclear |
|  | Mixed (any combination of the above; please specify) |
|  | Other (please specify) |
| What type(s) of intervention(s) was/were studied? Please choose ONE only | Pharmacological |
|  | Non-pharmacological (i.e. any intervention that does not involve drugs, e.g. device, behavioural, organisational, vitamins) |
|  | Both pharmacological and non-pharmacological |
| *What is the source of funding of the SR? Please choose ONE only | Non-profit sponsor (e.g. government, university/hospital/research institute, charitable foundation) |
|  | For-profit sponsor (e.g. pharmaceutical company) |
|  | Mixed (both for-profit and non-profit sponsors) |
|  | Unclear (do not know if the sponsor is for-profit or non-profit); please specify funder |
|  | Authors specified there was no funding for the SR |
|  | Not reported |
| Is a DOI for the SR report provided somewhere in the article? (e.g. doi:10.1002/14651858.MR000035.pub2) | Yes |
|  | No |
| Did the authors report registering this review? | Yes |
|  | No |
| What is the name of the register? | PROSPERO |
|  | Joanna Briggs Institute |
|  | Other (please specify) |
|  | Not reported |
| Is a registration number provided? | Yes |
|  | No |
| Is a hyperlink to the full registration record provided? | Yes |
|  | No |
| Where is the registration information listed in the report? | Title |
|  | Abstract |
|  | Introduction/Background |
|  | Methods |
|  | Results |
|  | Other special heading (please state) |
|  | Other location (please describe) |
| *Did the authors report working from a protocol or a priori established methods? There is no need to do an internet search for a protocol to answer this question | Yes, and protocol is publicly available (authors cite the bibliographic reference for the published protocol in the main text) |
|  | Yes, and protocol is publicly available (authors cite a website where the protocol can be accessed) |
|  | Yes, but protocol is not publicly available (i.e. authors state that they worked from a protocol but do not report the location of the protocol, or only report that it is available on request) |
|  | Unclear - the use of a protocol was only implied (e.g. by the term 'pre-specified'), please describe why you think a protocol was used: |
|  | No |
| *Which reporting guideline (if any) did the authors report using to guide the conduct or reporting of the review? Note that ‘PRISMA’ or ‘QUOROM’ should only be selected if the authors report that they conducted or reported their review according to either of these reporting guidelines. That is, neither guideline should be selected just because the authors presented a PRISMA or QUOROM flowchart (because authors may have created the flowchart but not referred to any other items in the PRISMA/QUOROM checklist). | PRISMA (Preferred Reporting Items for Systematic reviews and Meta-Analyses) |
|  | QUOROM (QUality of Reporting Of Meta-analyses) |
|  | MOOSE (Meta-analysis Of Observational Studies in Epidemiology) |
|  | MECIR (Methodological Expectation of Cochrane Intervention Reviews) |
|  | CAMARADES (Collaborative Approach to Meta-Analysis and Review of Animal Data from Experimental Studies) |
|  | Other (specify any other source specifically referred to as reporting guidance used) |
|  | None |
| Did the authors state that the SR was designed/conducted or reported according to the reporting guideline? | Authors state that the SR was DESIGNED/CONDUCTED/PERFORMED according to the reporting guideline |
|  | Authors state that the SR was REPORTED according to the reporting guideline |
|  | Both of the above |
|  | Other |
| Which of the following headings (or similar) are listed in the methods section of the manuscript? Check all that apply | “Search strategy” or “Data sources” |
|  | “Eligibility criteria” or “Study selection” |
|  | “Data extraction” |
|  | “Quality assessment” or “Risk of bias assessment” |
|  | “Data/statistical analysis” |
| *Did the authors report using Cochrane review methods? | Yes - this is a Cochrane review |
|  | Yes (for non-Cochrane reviews) – authors made a general statement such as "We conducted our review in accordance with methods recommended by the Cochrane Collaboration/Cochrane Handbook" |
|  | Yes (for non-Cochrane reviews) – authors reported that a particular review method (e.g. searching, risk of bias assessment, meta-analysis) was guided by Cochrane methods/guidance |
|  | No such statement was made (for non-Cochrane reviews) |
| *Did the authors make a statement regarding eligibility of studies based on their publication status? | Yes - authors stated both published and unpublished studies were eligible for inclusion (or that no restrictions on publication status were imposed) |
|  | Yes - authors stated only published studies were eligible for inclusion |
|  | Yes - authors stated only unpublished studies were eligible for inclusion |
|  | No such statement was made |
| *Did the authors make a statement regarding eligibility of studies based on language of publication? | Yes |
|  | No |
| *What languages were reported as being eligible for inclusion in this review? | English only |
|  | Language(s) other than English only (please specify all included languages) |
|  | Mixed: English and a specific language(s) other than English (please specify all other included languages) |
|  | All languages were considered - no restrictions |
| *Did the authors make a statement regarding eligibility of studies based on study design? | Yes - only the ELIGIBLE study designs were reported (e.g. "We only selected RCTs for inclusion") |
|  | Yes - only the INELIGIBLE (or excluded) study designs were reported (e.g. "Cohort studies and case series were excluded") |
|  | Yes - BOTH the eligible and ineligible study designs were reported (e.g. "We included RCTs and quasi-RCTs. Case-control studies were excluded") |
|  | No such statement was made |
| *Which study designs were eligible for inclusion in the review? Check the box if the authors stated in the Methods section that a particular design was eligible for inclusion, or if studies with a particular design were clearly included in the review (as identified in the Results section) [Check all that apply] | Randomized controlled trials (RCTs) |
|  | Quasi-randomized controlled trials |
|  | Other controlled experimental studies [i.e. non-randomized controlled trials (e.g. where allocation occurs in the course of usual treatment decisions or based on participants' choice), controlled before-and-after studies, interrupted time series studies] |
|  | Observational - cohort studies |
|  | Observational - case-control studies |
|  | Observational - cross-sectional studies |
|  | Observational - case studies or case series |
|  | Diagnostic accuracy studies (only tick if reviewers explicitly use this term) |
|  | Prognostic or predictive accuracy studies (only tick if reviewers explicitly use these terms) |
|  | Reviews/systematic reviews/meta-analyses |
|  | Other (please specify all others) |
|  | Unclear/not stated |
| How many electronic bibliographic databases were searched? [clinical trials registries (e.g. WHO ICTRP, www.clinicaltrials.gov) are NOT applicable to this question and so should not be counted here] |  |
| Which of the following electronic bibliographic databases were searched? [clinical trials registries (e.g. WHO ICTRP, www.clinicaltrials.gov) are NOT applicable to this question and so should not be counted here] | MEDLINE |
|  | PubMed |
|  | EMBASE (or Exerpta Medica) |
|  | Cochrane Central Register of Controlled Trials (CENTRAL) [do NOT tick if authors only report "Cochrane Library"] |
|  | "Cochrane Library" or other databases in The Cochrane Library (e.g. Cochrane Database of Systematic Reviews (CDSR) or DARE or HTA or NHSEED or Cochrane Methodology Register) |
|  | CINAHL |
|  | PsycInfo |
|  | Science Citation Index |
|  | Web of Science or Web of Knowledge (or ISI Web of Science or ISI Web of Knowledge) |
|  | SCOPUS |
|  | Google Scholar |
|  | Other (please specify) |
|  | No electronic bibliographic databases reported |
| *Were the years of coverage reported for electronic bibliographic databases? | Yes - both start and end dates are reported for ALL databases [Select this option if actual dates are reported, or if reviewers stated that they searched each database "from inception" or placed no limit/restriction on the start date, and reported the date the search was conducted] |
|  | Partially - start and end dates are reported for only one of many databases, or only the end date is reported for all databases (e.g. "we searched MEDLINE to May 2013") |
|  | No - not reported for any database |
| *Were search terms reported for one or more of the electronic databases? | Yes - Full Boolean search logic was reported for one or more database |
|  | Yes - Only main index terms (e.g. MeSH) were reported |
|  | Yes - Only free text words were reported |
|  | Yes - Both main index terms and free text words were listed, but no full Boolean search logic was reported |
|  | No - Readers are referred elsewhere for full search strategy |
|  | No - No search terms were reported |
| *Did the authors report searching at least one study registry to identify ongoing or completed studies? (a study "registry” refers to a database where trials are registered prior to recruitment of participants, e.g. WHO ICTRP, www.clinicaltrials.gov) | Yes |
|  | No |
| Which clinical trials or study registries were searched? Check all that apply | ClinicalTrials.gov |
|  | World Health Organization International Clinical Trials Registry Platform (WHO ICTRP) |
|  | www.controlled-trials.com |
|  | Other (please specify) |
| Which (if any) of the following other search methods did the authors report using? Check all that apply | Searching grey literature database (e.g. OpenSIGLE, OpenGrey) |
|  | Reviewing reference lists of relevant studies, reviews, or textbooks |
|  | Hand searching particular journal(s) |
|  | Reviewing abstracts/proceedings of specific conference(s) |
|  | Contacting experts or corresponding authors of included studies |
|  | Contacting a drug or device manufacturer |
|  | Contacting a regulator (e.g. Food and Drug Administration (FDA), European Medicines Agency (EMA)) |
|  | Other (please specify) |
|  | No other search methods were reported |
| *What method of study screening did the authors report using? | All titles/abstracts and full text articles were screened by two reviewers independently |
|  | All titles/abstracts and full text articles were screened using liberal acceleration (i.e. two reviewers needed to independently exclude a record, but only one reviewer needed to include a record) |
|  | All titles/abstracts and full text articles were screened by one reviewer, and a second reviewer screened a sample of records |
|  | All titles/abstracts and full text articles were screened by only one reviewer |
|  | Different method applied to titles/abstracts and full text articles (e.g. titles/abstracts screened using liberal acceleration, full text articles screened by two reviewers independently) |
|  | Two reviewers screened records for eligibility, but authors did not specify whether this method was applied independently to both titles/abstracts AND full text articles |
|  | Not reported |
|  | Other (please specify) |
| *What method of data extraction did the authors report using? | Two reviewers independently extracted data from all studies |
|  | Two reviewers extracted data from all studies, but authors did not state whether extraction was done independently |
|  | One reviewer extracted data from all studies, and another reviewer independently extracted data from a sample of studies |
|  | One reviewer extracted data from all studies, and another reviewer checked/verified the extracted data for all (or a sample of) studies |
|  | Only one reviewer extracted data from all studies (with no verification by another reviewer) |
|  | Not reported |
|  | Other (please specify) |
|  | Not applicable (e.g. no eligible studies identified) |
| Did the authors report assessing the risk of bias (or quality) of the included studies? Answer “Yes” regardless of the method used (i.e. does not have to have been assessed using an established tool) | Yes |
|  | No |
|  | Not applicable (e.g. no eligible studies identified) |
| *What method of risk of bias (or quality) assessment did the authors report using? | Two reviewers independently assessed all studies |
|  | Two reviewers assessed all studies, but authors did not state whether assessment was done independently |
|  | One reviewer assessed all studies, and another reviewer independently assessed a sample of studies |
|  | One reviewer assessed all studies, and another reviewer checked/verified the assessments for all (or a sample of) studies |
|  | Only one reviewer assessed all studies (with no verification by another reviewer) |
|  | Not reported |
|  | Other (please specify) |
| *Which risk of bias (or quality) assessment tool(s) did the authors report using? Check all that apply | Cochrane risk of bias tool (or modification) |
|  | Jadad Scale (or modification) |
|  | Newcastle-Ottawa Scale (or modification) |
|  | QUADAS or QUADAS-2 |
|  | Reporting guideline (e.g. CONSORT, STROBE, STARD) |
|  | Self-developed tool (i.e. developed by the review authors) |
|  | Other (please specify) |
|  | Not reported |
| Did the authors report assessing selective reporting (of outcomes or analyses, or both) in the included studies? | Yes |
|  | No |
|  | Not applicable (e.g. no eligible studies identified) |
| Did the authors report whether or not they contacted (or attempted to contact) corresponding authors of included studies for any unpublished data (e.g. unreported outcomes, unreported information required for risk of bias assessment)? | Yes - Review authors reported that they contacted (or attempted to contact) authors of included studies |
|  | Yes - Review authors reported that they DID NOT contact authors of included studies |
|  | No such statement was made |
|  | Not applicable (e.g. no eligible studies identified) |
| *Was the review flow (i.e. a description of the number of records screened and included/excluded) reported in the review? | Yes - review flow was reported both in text/table AND in a PRISMA/QUOROM-like flow diagram |
|  | Yes - review flow was only reported in a PRISMA/QUOROM-like flow diagram |
|  | Yes - review flow was only reported in text/table |
|  | No - review flow was not reported |
| *Were the reasons for the exclusion of studies from the systematic review reported? Answer this question only in relation to full text articles retrieved | Yes - reasons for exclusion of ALL excluded full text articles were reported both in text/table AND in a PRISMA/QUOROM-like flow diagram |
|  | Yes - reasons for exclusion of ALL excluded full text articles were only reported in a PRISMA/QUOROM-like flow diagram |
|  | Yes - reasons for exclusion of ALL excluded full text articles were only reported in text/table |
|  | Partially - reasons for exclusion of only SOME excluded full text articles were reported |
|  | No - none of the full text articles retrieved were excluded |
|  | No - reasons for exclusion were not reported for any of the excluded full text articles |
| *What was the total number of records retrieved across all searches (either electronic or other)? Enter either the reported number, 'NR' if not reported, or 'U' if unclear. If SR is an updated SR, report total number rather than separate numbers for previous and updated search |  |
| *What was the number of unique records (i.e. after duplicates removed) retrieved from all searches (either electronic or manual)? Enter either the reported number, 'NR' if not reported, or 'U' if unclear. If SR is an updated SR, report total number rather than separate numbers for previous and updated search |  |
| *What was the number of titles/abstracts screened after duplicate references were removed? (this number is likely to be the same as the number entered for the preceding item, though in some cases may not). Enter either the reported number, 'NR' if not reported, or 'U' if unclear. If SR is an updated SR, report total number rather than separate numbers for previous and updated search |  |
| *What was the number of full text articles sought for more detailed evaluation by reviewers? Enter either the reported number, 'NR' if not reported, or 'U' if unclear. If SR is an updated SR, report total number rather than separate numbers for previous and updated search |  |
| *What was the number of REPORTS (rather than studies) included in the systematic review? (e.g. a study may have been published as a conference abstract and 3 journal articles, thus yielding 4 reports). Enter either the reported number, 'NR' if not reported, or 'U' if unclear. If SR is an updated SR, report total number rather than separate numbers for previous and updated search |  |
| *What was the number of STUDIES (rather than reports) included in the systematic review? Enter either the reported number, 'NR' if not reported, or 'U' if unclear. If SR is an updated SR, report total number rather than separate numbers for previous and updated search |  |
| Are the included studies cited/listed within the main manuscript text (i.e. in Results section text or in a table)? | Yes |
|  | No |
|  | Not applicable (e.g. no eligible studies identified) |
| Under which of the following sections are included studies cited/listed within the main manuscript text? (select all that apply) | Methods section |
|  | Results section (text) |
|  | Results section (table) |
|  | Study flow diagram |
|  | Other (please specify) |
| Does the word “included”, the phrase “included study/studies”, or some other relevant descriptor precede the listing or description of included studies in the main text? | Yes |
|  | No |
| If applicable, copy and paste the heading, subheading, and actual phrasing preceding the listing of included studies in the main manuscript text: (please only select the options that apply) | Heading: |
|  | Subheading: |
|  | Any descriptive text (“eligible” or “included” studies): |
| Are citations for included studies hyperlinked to a bibliographic reference list? | Yes |
|  | No |
| Are the DOIs for the citations of all included studies provided in the reference list? | Yes |
|  | No |
| Are references for the included studies listed within the main reference list or separately from other references? | Listed within the main reference list (i.e. not distinguished from other references) |
|  | Listed in a separate reference list |
|  | References for the included studies were not reported |
| *Did the authors report that any type of grey literature was included in the systematic review? By grey literature we mean internal reports (e.g. from pharmaceutical companies); conference abstracts/proceedings; symposia/workshop reports/proceedings of meetings; Doctoral/Masters dissertations; personal observations (no official presentation or publication/informal communications); census, economic and other government reports; data from databases of ongoing research; or newsletters. Note that to answer “Yes”, the review authors do NOT have to have used the term “grey literature”. They could just state something like “We included 3 conference abstracts” or “We included a dissertation”. | Yes - the authors reported that a type of grey literature was included in the systematic review |
|  | No such statement was made |
|  | Unclear |
| *What was the total number of participants in the systematic review? If this total number is not clearly stated in the report, type 'NR' (e.g. it is not necessary to add up sample sizes reported in a table). Enter either the reported number, 'NR' if not reported, or 'Unclear'. If the unit of analysis is body parts (e.g. eyes, knees), please specify this. |  |
| Which number of participants was reported in the abstract? | Both the total number of participants summed across all studies in the systematic review AND the number of participants included in at least one meta-analysis |
|  | Only the total number of participants summed across all studies in the systematic review |
|  | Only the number of participants included in at least one meta-analysis |
|  | No number of participants reported |
|  | Unclear |
|  | Other (please specify) |
|  | Not applicable (e.g. no eligible studies identified) |
| *Did the review authors specify in the Methods section the outcomes that were eligible for inclusion in the review? (e.g. listed outcomes under the eligibility criteria, or stated "We included studies measuring survival and response rate", or stated "We extracted/collected data on pain, quality of life...") | Yes |
|  | No |
| *How many review outcomes were specified in the METHODS section of the review? *Only count the number of outcome domains. An outcome domain is a true state or endpoint of interest, irrespective of how it is measured (e.g. presence or severity of depression). An outcome domain is different to an outcome measurement, which is a specific measurement made on the study participants (e.g. measurement of depression using the Hamilton rating scale 6 weeks after initiation of treatment). An outcome domain is also different to an outcome analysis, which is a specific result obtained by analysing one or more outcome measurements (e.g. the difference in mean change in Hamilton rating scale scores from baseline to 6 weeks between intervention and control groups) |  |
| *How many review outcomes were specified in the RESULTS section of the review? *Only count the number of outcome domains. An outcome domain is a true state or endpoint of interest, irrespective of how it is measured (e.g. presence or severity of depression). An outcome domain is different to an outcome measurement, which is a specific measurement made on the study participants (e.g. measurement of depression using the Hamilton rating scale 6 weeks after initiation of treatment). An outcome domain is also different to an outcome analysis, which is a specific result obtained by analysing one or more outcome measurements (e.g. the difference in mean change in Hamilton rating scale scores from baseline to 6 weeks between intervention and control groups) |  |
| Which measures of diagnostic accuracy were specified in the Methods section of the SR? Check all that apply | Sensitivity |
|  | Specificity |
|  | Positive predictive value (PPV) |
|  | Negative predictive value (NPV) |
|  | Likelihood ratios |
|  | Area under the ROC curve (AUC) |
|  | Diagnostic odds ratio (DOR) |
|  | Other (please specify) |
| *Did the review authors specify one or more primary outcome(s)? [Answer ‘Yes’ if only one outcome is specified in the review] | Yes |
|  | No |
|  | No but only one outcome reported |
|  | Not applicable (SR of diagnostic test accuracy) |
| *What type of outcome is the (1st) primary outcome? | Dichotomous |
|  | Continuous |
|  | Both dichotomous and continuous |
|  | Rate |
|  | Time-to-event |
|  | Other (please specify) |
|  | Unclear |
|  | Not reported |
| *What is the unit of measure of the first reported result (effect estimate) of the primary outcome? Note that the first result may be identified from the Abstract or Results section of the review, depending on where it is first reported in the publication | Mean difference |
|  | Standardized mean difference |
|  | Risk ratio |
|  | Odds ratio |
|  | Risk difference |
|  | Hazard ratio |
|  | Likelihood ratios |
|  | Prevalence |
|  | Other (please specify) |
|  | Unclear |
|  | Not reported |
| *What is the statistical significance of the first reported result (effect estimate) of the primary outcome? Note that the first result may be identified from the Abstract or Results section of the review, depending on where it is first reported in the publication. If it is unclear which is the “intervention” and which is the “comparator” (e.g. because the authors compare two interventions e.g. drug dose A versus drug dose B, or manual therapy versus exercise, WITHOUT specifying which one is the “active” intervention, select “Unclear” | Favourable, statistically significant (i.e. effect in favour of the intervention with p ≤ 0.05) |
|  | Favourable, non-statistically significant (i.e. effect in favour of the intervention with p > 0.05) |
|  | Unfavourable, statistically significant (i.e. effect in favour of the comparator with p ≤ 0.05) |
|  | Unfavourable, non-statistically significant (i.e. effect in favour of the comparator with p > 0.05) |
|  | Non-comparative (e.g. review of prevalence) |
|  | Unclear |
|  | Not reported |
| Were two or more studies synthesized statistically (i.e. in a meta-analysis)? | Yes |
|  | No |
|  | Not applicable (< 2 included studies) |
| Which meta-analysis model was used in the meta-analyses? | Fixed-effect model for all meta-analyses |
|  | Random-effects model for all meta-analyses |
|  | Varied i.e. fixed-effect model for some meta-analyses and random-effects model for other meta-analyses |
|  | Other (please specify) |
|  | Not reported |
| *How many studies were included in the largest meta-analysis? |  |
| *Was the risk of bias (or quality) assessment incorporated into ANY meta-analyses in the review (e.g. authors restricted meta-analyses to low risk of bias studies only, or investigated the impact of study risk of bias using sensitivity analyses, subgroup analyses, meta-regression, bias modelling, or “quality-effects” model)? | Yes |
|  | No |
|  | Not applicable - no risk of bias (or quality) assessment |
| Was any method described to formally evaluate statistical heterogeneity of included studies? | Yes |
|  | No |
|  | Statistical heterogeneity was not taken into account using formal statistical evaluation, but heterogeneity of the studies was qualitatively assessed (e.g. in some form of narrative discussion) |
| Which methods were used to formally evaluate statistical heterogeneity of included studies? [Check all that apply] | Visual inspection of the forest plot |
|  | L'Abbe plot |
|  | Chi-square or Cochran's Q |
|  | I^2 (I-square) |
|  | tau^2 (tau-square) |
|  | Other (please specify) |
| *Did the authors report that a measure of statistical heterogeneity (e.g. Chi-square, I-square, tau-square or any other) was used to justify use of a fixed-effect or random-effects meta-analysis model (e.g. stated something like “If I-square was above 50% we used the random-effects model; if less than 50% we used the fixed-effect model)? | Yes |
|  | No |
| Did the authors report assessing (or an intent to assess) publication bias? | Yes, publication bias was assessed |
|  | No, publication bias was not assessed, but the authors reported that they intended to assess it if they identified a sufficient number of studies |
|  | No, publication bias was not assessed, and the authors did not report an intention to assess it |
| Which methods did the authors report using (or intending to use) to assess publication bias? Check all that apply | Funnel plot |
|  | Rank correlation |
|  | Trim and fill method |
|  | Egger's test |
|  | Begg's test |
|  | Selection model |
|  | Fail-safe method |
|  | Subgroup analyses by sample size |
|  | Sensitivity analysis comparing fixed-effect to random-effects model |
|  | Other (please specify) |
| *Did the authors discuss the results in relation to potential publication bias? Rate as “Yes” if the authors considered the possible impact that publication bias may have had on the results, regardless of whether they could formally assess publication bias (e.g. because there were fewer than 10 included studies) | Yes |
|  | No |
| Which of the following additional analyses did the authors conduct? Check all that apply | Subgroup analysis |
|  | Sensitivity analysis |
|  | Meta-regression |
|  | Network meta-analysis (also referred to as indirect comparisons, mixed-treatment comparisons, or multiple-treatments meta-analysis) |
|  | Individual patient data (IPD) meta-analysis |
|  | Other (please specify) |
|  | No additional analyses |
| *Did the authors report an analysis of harms/adverse events (or an intent to analyse harms/adverse events) as part of this systematic review? | Yes - data on harms/adverse events were reported |
|  | No - authors reported a plan to analyse harms/adverse events but did not identify any eligible studies, or none of the included studies measured harms/adverse events |
|  | No - authors did not report any harms/adverse events data or any plan to analyse harms/adverse events |
|  | Not applicable (SR of diagnostic test accuracy, prognosis or prevalence) |
| *Did the authors report an analysis of cost effectiveness (or an intent to analyse cost effectiveness) as part of this systematic review? | Yes - cost-effectiveness data were reported |
|  | No - authors reported a plan to analyse cost-effectiveness but did not identify any eligible studies, or none of the included studies measured cost-effectiveness |
|  | No - authors did not report any cost-effectivness data or any plan to analyse cost-effectiveness |
|  | Not applicable (SR of diagnostic test accuracy, prognosis or prevalence) |
| *Did the authors report an assessment of the quality of evidence using the GRADE approach? | Yes – authors reported a GRADE assessment in a Summary of Findings table |
|  | Yes – authors reported a GRADE assessment in text only |
|  | No – authors reported an intent to perform a GRADE assessment but did not identify any eligible studies |
|  | No – authors did not report performing, or an intent to perform, a GRADE assessment |
|  |  |
| *Were any limitations reported in the Discussion section? Only answer "Yes" if the authors used terms such as "limitation", "limited" or "flawed" when describing a particular method | Yes – both limitations at the study level (e.g. risk of bias of included studies) and review level (e.g. incomplete retrieval of identified research, reporting bias) were reported |
|  | Yes – only limitations at the study level were reported |
|  | Yes – only limitations at the review level were reported |
|  | No limitations were reported |
| Was the risk of bias/quality/limitations of the included studies incorporated into the abstract conclusion? | Yes - incorporated |
|  | No – not incorporated (the authors did not acknowledge any limitations throughout the review, or acknowledged some limitations in the Results/Discussion yet ignored this when reporting the abstract conclusion, e.g. state that an intervention works or should be recommended for routine practice, without noting that the evidence on which this conclusion is based was of poor quality) |
| Did review authors declare whether they had any conflicts of interest? | Yes |
|  | No |
| Did review authors report whether authors of the included studies had any conflicts of interest, or report the funding source of the included studies? | Yes |
|  | No |
| General notes/comments |  |
