# Supplementary material for: Epidemiology and Reporting Characteristics of Systematic Reviews of Biomedical Research: A Cross-Sectional Study
Source: PLoS Med. 2016 May 24;13(5):e1002028. doi: 10.1371/journal.pmed.1002028 (PMC4878797; doi:10.1371/journal.pmed.1002028)
Supplement: S1 Results — (DOCX) [file pmed.1002028.s003.docx]

**Supplementary Results**

**Calculating the annual prevalence of SRs**

In the 2004 sample, 300 SRs were identified. Of these, 125 were Cochrane reviews and 175 were non-Cochrane SRs. Cochrane reviews were published on a quarterly basis in 2004. Therefore, to estimate the annual prevalence in 2004, the following calculation was performed: (125 x 4) + (175 x 12) = 2,600

In the 2014 sample, 682 SRs were identified. Cochrane reviews were published on a monthly basis in 2014, so to estimate the annual prevalence in 2014, the following calculation was performed: 682 x 12 = 8184

The relative increase in annual SR prevalence from 2004 to 2014 was calculated as

8184/2600 = 3.1 (i.e. a 3-fold increase).

**Supplementary Table 1. Reporting characteristics of systematic reviews indexed in February 2014 (complete table of results)**

| **Category** | **Characteristic** | **Overall**  **Number (%), of n = 300**^a^ | **Therapeutic (Cochrane)**  **Number (%), of n = 45**^a^ | **Therapeutic (non-Cochrane)**  **Number (%), of n = 119**^a^ | **Epidemiology**  **Number (%), of n = 74**^a^ | **Diagnosis / Prognosis**  **Number (%), of n = 33**^a^ | **Other**  **Number (%), of n = 29**^a^ |
| --- | --- | --- | --- | --- | --- | --- | --- |
| Terms used in the title or abstract of the SR | “Systematic review” only | 85 (28) | 0 (0) | 34 (29) | 21 (28) | 9 (27) | 21 (72) |
|  | “Meta-analysis” only | 89 (30) | 15 (33) | 37 (31) | 25 (34) | 11 (33) | 1 (3) |
|  | Both “systematic review” and “meta-analysis” | 80 (27) | 0 (0) | 42 (35) | 23 (31) | 12 (36) | 3 (10) |
|  | Neither | 46 (15) | 30 (67) | 6 (5) | 5 (7) | 1 (3) | 4 (14) |
| Registration of SR mentioned | Registered in a database (e.g. PROSPERO) | 12 (4) | 0 (0) | 8 (7) | 3 (4) | 1 (3) | 0 (0) |
|  | Not registered | 288 (96) | 45 (100) | 111 (93) | 71 (96) | 32 (97) | 29 (100) |
| SR protocol mentioned | Protocol is publicly available | 49 (16) | 44 (98) | 5 (4) | 0 (0) | 0 (0) | 0 (0) |
|  | Protocol mentioned, but not publicly available | 28 (9) | 0 (0) | 21 (18) | 3 (4) | 2 (6) | 2 (7) |
|  | Unclear (the use of a protocol was only implied) | 14 (5) | 0 (0) | 5 (4) | 5 (7) | 4 (12) | 0 (0) |
|  | Not mentioned | 209 (70) | 1 (2) | 88 (74) | 66 (89) | 27 (82) | 27 (93) |
| Reporting guideline mentioned (other than just presenting PRISMA flow diagram) | PRISMA | 68 (23) | 0 (0) | 38 (32) | 16 (22) | 5 (15) | 9 (31) |
|  | QUOROM | 2 (1) | 0 (0) | 1 (1) | 0 (0) | 1 (3) | 0 (0) |
|  | MOOSE | 13 (4) | 0 (0) | 3 (3) | 8 (11) | 2 (6) | 0 (0) |
|  | MECIR | 1 (1) | 1 (2) | 0 (0) | 0 (0) | 0 (0) | 0 (0) |
|  | Other (e.g. both PRISMA and MOOSE) | 3 (1) | 0 (0) | 0 (0) | 3 (4) | 0 (0) | 0 (0) |
|  | None | 213 (71) | 44 (98) | 77 (65) | 47 (64) | 25 (76) | 20 (69) |
| How reporting guideline was used | To guide design/conduct of the SR | 45/87 (52) | 0 (0) | 24/42 (57) | 13/27 (48) | 5/8 (63) | 3/9 (33) |
|  | To guide reporting of the SR | 22/87 (25) | 1/1 (100) | 14/42 (33) | 2/27 (7) | 3/8 (38) | 2/9 (22) |
|  | Both of the above | 9/87 (10) | 0 (0) | 2/42 (5) | 5/27 (19) | 0 (0) | 2/9 (22) |
|  | Other (e.g. PRISMA checklist submitted as appendix but not mentioned elsewhere, or stated that PRISMA “was followed”) | 11/87 (13) | 0 (0) | 2/42 (5) | 7/27 (26) | 0 (0) | 2/9 (22) |
| Cochrane methods used | Stated that Cochrane methods informed conduct of at least one component the SR | 138 (46) | 45 (100) | 64 (54) | 16 (22) | 9 (27) | 4 (14) |
|  | No reference to Cochrane methods | 162 (54) | 0 (0) | 55 (46) | 58 (78) | 24 (73) | 25 (86) |
| Eligible publication status | Both published and unpublished studies | 116 (39) | 41 (91) | 49 (41) | 13 (18) | 6 (18) | 7 (24) |
|  | Only published studies | 80 (27) | 2 (4) | 33 (28) | 25 (34) | 9 (27) | 11 (38) |
|  | Only unpublished studies | 1 (1) | 0 (0) | 1 (1) | 0 (0) | 0 (0) | 0 (0) |
|  | Not reported | 103 (34) | 2 (4) | 36 (30) | 36 (49) | 18 (55) | 11 (38) |
| Eligible languages | All languages considered | 129 (43) | 37 (82) | 48 (40) | 29 (39) | 10 (30) | 5 (17) |
|  | English only | 92 (31) | 1 (2) | 44 (37) | 22 (30) | 12 (36) | 13 (45) |
|  | Mixed (English and a specific LOE) | 31 (10) | 1 (2) | 9 (8) | 10 (14) | 5 (15) | 6 (21) |
|  | Only LOE | 0 (0) | 0 (0) | 0 (0) | 0 (0) | 0 (0) | 0 (0) |
|  | Not reported | 48 (16) | 6 (13) | 18 (15) | 13 (18) | 6 (18) | 5 (17) |
| Eligibility criteria for study designs | Both eligible and ineligible study designs reported | 69 (23) | 13 (29) | 33 (28) | 17 (23) | 4 (12) | 2 (7) |
|  | Only eligible study designs reported | 144 (48) | 32 (71) | 67 (56) | 32 (43) | 5 (15) | 8 (28) |
|  | Only ineligible study designs reported | 24 (8) | 0 (0) | 4 (3) | 7 (9) | 8 (24) | 5 (17) |
|  | Not reported | 63 (21) | 0 (0) | 15 (13) | 18 (24) | 16 (48) | 14 (48) |
| Eligible study designs | Randomized controlled trials (RCTs) | 158 (53) | 44 (98) | 99 (83) | 7 (9) | 1 (3) | 7 (24) |
|  | Quasi-randomized controlled trials | 33 (11) | 14 (31 ) | 15 (13) | 3 (4) | 0 (0) | 1 (3) |
|  | Other controlled experimental studies (e.g. non-randomized controlled trial, controlled before-and-after study, interrupted time series study) | 30 (10) | 4 (9) | 18 (15) | 5 (7) | 1 (3) | 2 (7) |
|  | Observational - cohort studies | 76 (25) | 0 (0) | 25 (21) | 37 (50) | 10 (30) | 4 (14) |
|  | Observational - case-control studies | 49 (16) | 0 (0) | 8 (7) | 37 (50) | 2 (6) | 2 (7) |
|  | Observational - cross-sectional studies | 31 (10) | 0 (0) | 10 (8) | 17 (23) | 0 (0) | 4 (14) |
|  | Observational - case studies or case series | 19 (6) | 0 (0) | 10 (8) | 6 (8) | 2 (6) | 1 (3) |
|  | Other (e.g. observational studies not specified, qualitative studies) | 56 (19) | 1 (2) | 22 (18) | 11 (15) | 8 (24) | 14 (48) |
|  | Unclear/not stated | 36 (12) | 0 (0) | 3 (3) | 7 (9) | 16 (48) | 10 (34) |
|  | Restricted to RCTs and quasi-RCTs | 107 (36) | 40 (89) | 64 (54) | 1 (1) | 1 (3) | 1 (3) |
| Number of databases searched | Median (IQR) | 4 (3-5) | 5 (4-6) | 3 (2-5) | 3 (2-5) | 3 (2-3) | 5 (3-6) |
|  | Only 1 database searched | 28 (9) | 0 (0) | 11 (9) | 13 (18) | 1 (3) | 3 (10) |
| Databases searched | MEDLINE/PubMed | 294 (98) | 44 (98) | 117 (98) | 73 (99) | 33 (100) | 27 (93) |
|  | EMBASE | 208 (69) | 39 (87) | 82 (69) | 44 (59) | 27 (82) | 16 (55) |
|  | Cochrane Central Register of Controlled Trials (CENTRAL) | 95 (32) | 40 (89) | 46 (39) | 3 (4) | 4 (12) | 2 (7) |
|  | “Cochrane Library” or other databases in The Cochrane Library | 81 (27) | 2 (4) | 45 (38) | 15 (20) | 12 (36) | 7 (24) |
|  | CINAHL | 65 (22) | 15 (33) | 27 (23) | 10 (14) | 3 (9) | 10 (34) |
|  | PsycInfo | 49 (16) | 10 (22) | 18 (15) | 12 (16) | 0 (0) | 9 (31) |
|  | Science Citation Index | 12 (4) | 6 (13) | 4 (3) | 2 (3) | 0 (0) | 0 (0) |
|  | Web of Science or Web of Knowledge | 63 (21) | 6 (13) | 17 (14) | 26 (35) | 6 (18) | 8 (28) |
|  | SCOPUS | 29 (10) | 2 (4) | 15 (13) | 5 (7) | 2 (6) | 5 (17) |
|  | Google Scholar | 19 (6) | 1 (2) | 10 (8) | 4 (5) | 1 (3) | 3 (10) |
|  | Other (e.g. AMED, CNKI, ERIC, LILACS) | 133 (44) | 36 (80) | 37 (31) | 34 (46) | 9 (27) | 17 (59) |
| Years of coverage reported | Both start and end dates are reported for all databases | 196 (65) | 41 (91) | 78 (66) | 39 (53) | 19 (58) | 19 (66) |
|  | Partially - start and end dates are reported for only one of many databases, or only the end date is reported for all databases | 88 (29) | 4 (9) | 35 (29) | 30 (41) | 13 (39) | 6 (21) |
|  | Not reported for any database | 16 (5) | 0 (0) | 6 (5) | 5 (7) | 1 (3) | 4 (14) |
| Search terms reported | Full Boolean search logic reported for one or more database | 134 (45) | 44 (98) | 41 (34) | 26 (35) | 13 (39) | 10 (34) |
|  | Only main index terms (e.g. MeSH) reported | 12 (4) | 0 (0) | 6 (5) | 4 (5) | 1 (3) | 1 (3) |
|  | Only free text words reported | 114 (38) | 0 (0) | 54 (45) | 34 (46) | 14 (42) | 12 (41) |
|  | Both main index terms and free text words listed, but no full Boolean search logic reported | 24 (8) | 0 (0) | 11 (9) | 7 (9) | 4 (12) | 2 (7) |
|  | Readers are referred elsewhere for full search strategy (other than online appendix) | 10 (3) | 1 (2) | 6 (5) | 1 (1) | 0 (0) | 2 (7) |
|  | No search terms were reported | 6 (2) | 0 (0) | 1 (1) | 2 (3) | 1 (3) | 2 (7) |
| Trial registry searched | At least one register searched (e.g. ClinicalTrials.gov) | 58 (19) | 28 (62) | 24 (20) | 4 (5) | 2 (6) | 0 (0) |
| Number of other sources searched | Median (IQR) | 1 (1-2) | 2 (1-3) | 1 (1-2) | 1 (1-1) | 1 (1-1) | 1 (1-2) |
| Other sources searched | Grey literature database (e.g. OpenSIGLE) | 21 (7) | 9 (20) | 8 (7) | 1 (1) | 2 (6) | 1 (3) |
|  | Reviewing reference lists of relevant studies, reviews, or textbooks | 243 (81) | 38 (84) | 99 (83) | 58 (78) | 27 (82) | 21 (72) |
|  | Hand searching particular journal(s) | 25 (8) | 6 (13) | 12 (10) | 2 (3) | 1 (3) | 4 (14) |
|  | Reviewing abstracts/proceedings of specific conference(s) | 47 (16) | 11 (24) | 26 (22) | 7 (9) | 1 (3) | 2 (7) |
|  | Contacting experts or corresponding authors of included studies | 54 (18) | 23 (51) | 16 (13) | 8 (11) | 5 (15) | 2 (7) |
|  | Contacting a drug or device manufacturer | 11 (4) | 8 (18) | 3 (3) | 0 (0) | 0 (0) | 0 (0) |
|  | Contacting a drug or device regulator (e.g. Food and Drug Administration (FDA), European Medicines Agency (EMA)) | 2 (1) | 0 (0) | 2 (2) | 0 (0) | 0 (0) | 0 (0) |
|  | Other (e.g. citation tracking, personal files) | 35 (12) | 8 (18) | 13 (11) | 5 (7) | 6 (18) | 3 (10) |
|  | No other search methods reported | 40 (13) | 4 (9) | 13 (11) | 13 (18) | 4 (12) | 6 (21) |
| Screening method | Two reviewers independently screened all titles/abstracts and full text | 80 (27) | 22 (49) | 31 (26) | 15 (20) | 6 (18) | 6 (21) |
|  | Two reviewers screened records for eligibility, but unclear if this method was applied independently to both titles/abstracts AND full text articles | 86 (29) | 15 (33) | 32 (27) | 19 (26) | 11 (33) | 9 (31) |
|  | One reviewer screened all titles/abstracts and full text articles, and a second reviewer screened a sample of records | 7 (2) | 1 (2) | 3 (3) | 3 (4) | 0 (0) | 0 (0) |
|  | Only one reviewer screened all titles/abstracts and full text articles | 6 (2) | 0 (0) | 3 (3) | 1 (1) | 0 (0) | 2 (7) |
|  | All titles/abstracts and full text articles screened using liberal acceleration | 2 (1) | 0 (0) | 0 (0) | 1 (1) | 0 (0) | 1 (3) |
|  | Different method applied to titles/abstracts and full text articles | 18 (6) | 5 (11) | 1 (1) | 5 (7) | 5 (15) | 2 (7) |
|  | Other (e.g. screened by 3 authors) | 14 (5) | 2 (4) | 9 (8) | 1 (1) | 2 (6) | 0 (0) |
|  | Not reported | 87 (29) | 0 (0) | 40 (34) | 29 (39) | 9 (27) | 9 (31) |
| Data extraction method | Two reviewers independently extracted data from all studies | 131 (44) | 38 (84) | 37 (31) | 34 (46) | 16 (48) | 6 (21) |
|  | Two reviewers extracted data from all studies, but unclear if this was done independently | 21 (7) | 2 (4) | 8 (7) | 6 (8) | 3 (9) | 2 (7) |
|  | One reviewer extracted data from all studies, and another reviewer checked/verified the extracted data for all (or a sample of) studies | 29 (10) | 0 (0) | 15 (13) | 9 (12) | 1 (3) | 4 (14) |
|  | Only one reviewer extracted data from all studies | 7 (2) | 0 (0) | 2 (2) | 0 (0) | 2 (6) | 3 (10) |
|  | Other (e.g. extracted by 3 authors) | 11 (4) | 1 (2) | 4 (3) | 3 (4) | 3 (9) | 0 (0) |
|  | NA (no eligible studies identified) | 4 (1) | 3 (7) | 1 (1) | 0 (0) | 0 (0) | 0 (0) |
|  | Not reported | 97 (32) | 1 (2) | 52 (44) | 22 (30) | 8 (24) | 14 (48) |
| Study risk of bias/quality formally assessed | Yes | 206 (69) | 42 (93) | 87 (73) | 44 (59) | 22 (67) | 11 (38) |
|  | NA (no eligible studies identified) | 4 (1) | 3 (7) | 1 (1) | 0 (0) | 0 (0) | 0 (0) |
|  | No | 90 (30) | 0 (0) | 31 (26) | 30 (41) | 11 (35) | 18 (62) |
| Study risk of bias/quality assessment method | Two reviewers independently assessed all studies | 89/206 (43) | 30/42 (71) | 29/87 (33) | 17/44 (39) | 9/22 (41) | 4/11 (36) |
|  | Two reviewers assessed all studies, but authors did not state whether assessment was done independently | 16/206 (8) | 3/42 (7) | 7/87 (8) | 4/44 (9) | 1/22 (5) | 1/11 (9) |
|  | One reviewer assessed all studies, and another reviewer checked/verified the assessments for all (or a sample of) studies | 6/206 (3) | 0 (0) | 4/87 (5) | 2/44 (5) | 0 (0) | 0 (0) |
|  | Only one reviewer assessed all studies | 3/206 (1) | 0 (0) | 1/87 (1) | 1/44 (2) | 0 (0) | 1/11 (9) |
|  | Other (e.g. assessed by 3 authors) | 16/206 (8) | 4/42 (10) | 5/87 (6) | 3/44 (7) | 4/22 (18) | 0 (0) |
|  | Not reported | 76/206 (37) | 5/42 (12) | 41/87 (47) | 17/44 (39) | 8/22 (36) | 5/11 (45) |
| Study risk of bias/quality assessment tool used | Cochrane risk of bias tool (or modification) | 77/206 (37) | 37/42 (88) | 36/87 (41) | 4/44 (9) | 0 (0) | 0 (0) |
|  | Jadad Scale (or modification) | 17/206 (8) | 0 (0) | 17/87 (20) | 0 (0) | 0 (0) | 0 (0) |
|  | Newcastle-Ottawa Scale (or modification) | 17/206 (8) | 1/42 (2) | 6/87 (7) | 8/44 (18) | 1/22 (5) | 1/11 (9) |
|  | QUADAS or QUADAS-2 | 8/206 (4) | 0 (0) | 0 (0) | 0 (0) | 7/22 (32) | 1/11 (9) |
|  | Reporting guideline (e.g. CONSORT) | 15/206 (7) | 0 (0) | 9/87 (10) | 3/44 (7) | 2/22 (9) | 1/11 (9) |
|  | Tool developed by review authors | 38/206 (18) | 2/42 (5) | 9/87 (10) | 16/44 (36) | 8/22 (36) | 3/11 (27) |
|  | Other (please specify) | 53/206 (26) | 3/42 (7) | 21/87 (24) | 14/44 (32) | 8/22 (36) | 7/11 (64) |
|  | Not reported | 6/206 (3) | 0 (0) | 2/87 (2) | 2/44 (5) | 2/22 (9) | 0 (0) |
| Selective outcome/analysis reporting assessed | Yes | 70 (23) | 36 (80) | 30 (25) | 3 (4) | 1 (3) | 0 (0) |
|  | NA (e.g. no eligible studies identified) | 4 (1) | 3 (7) | 1 (1) | 0 (0) | 0 (0) | 0 (0) |
|  | No | 226 (75) | 6 (13) | 88 (74) | 71 (96) | 32 (97) | 29 (100) |
| Study authors contacted | Attempts made to contact authors | 99 (33) | 35 (78) | 33 (28) | 18 (24) | 8 (24) | 5 (17) |
|  | No attempt made to contact authors | 14 (5) | 4 (9) | 2 (2) | 2 (3) | 6 (18) | 0 (0) |
|  | NA (e.g. no eligible studies identified) | 4 (1) | 3 (7) | 1 (1) | 0 (0) | 0 (0) | 0 (0) |
|  | Not reported | 183 (61) | 3 (7) | 83 (70) | 54 (73) | 19 (58) | 24 (83) |
| Review flow reported | Reported in PRISMA-like flow diagram | 206 (69) | 23 (51) | 94 (79) | 46 (62) | 26 (79) | 17 (59) |
|  | Reported in text/table only | 20 (7) | 5 (11) | 5 (4) | 6 (8) | 2 (6) | 2 (7) |
|  | Partially reported | 38 (13) | 7 (16) | 14 (12) | 11 (15) | 2 (6) | 4 (14) |
|  | Not reported | 36 (12) | 10 (22) | 6 (5) | 11 (15) | 3 (9) | 6 (21) |
| Reasons for exclusion of full text articles reported | Reasons for all excluded articles reported in text/table or in PRISMA-like flow diagram (or both) | 199 (66) | 39 (87) | 82 (69) | 40 (54) | 25 (76) | 13 (45) |
|  | Partially - reasons for only some excluded articles reported | 28 (9) | 4 (9) | 9 (8) | 13 (18) | 1 (3) | 1 (3) |
|  | No full text articles retrieved were excluded | 12 (4) | 2 (4) | 5 (4) | 2 (3) | 3 (10) | 0 (0) |
|  | Not reported for any articles | 61 (20) | 0 (0) | 23 (19) | 19 (26) | 4 (12) | 15 (52) |
| Grey literature included | At least one type of grey literature (e.g. conference abstract, thesis) was included | 26 (9) | 8 (18) | 11 (9) | 2 (3) | 1 (3) | 4 (14) |
| Total number of included participants | Reported in main text | 194/296 (66) | 39/42 (93) | 83/118 (70) | 42/74 (57) | 26/33 (79) | 4/29 (14) |
|  | Reported in abstract | 147/296 (50) | 37/42 (88) | 56/118 (47) | 34/74 (46) | 17/33 (52) | 3/29 (10) |
| Number of participants reported in the abstract | Both the total number of participants summed across all studies in the systematic review and the number of participants included in at least one meta-analysis | 29 (10) | 18 (40) | 9 (8) | 1 (1) | 1 (3) | 0 (0) |
|  | Only the total number of participants summed across all studies in the systematic review | 118 (39) | 19 (42) | 47 (40) | 33 (45) | 16 (48) | 3 (10) |
|  | Only the number of participants included in at least one meta-analysis | 8 (3) | 4 (9) | 3 (3) | 1 (1) | 0 (0) | 0 (0) |
|  | Unclear | 1 (1) | 0 (0) | 1 (1) | 0 (0) | 0 (0) | 0 (0) |
|  | Other | 4 (1) | 0 (0) | 2 (2) | 1 (1) | 1 (3) | 0 (0) |
|  | NA (no eligible studies identified) | 4 (1) | 3 (7) | 1 (1) | 0 (0) | 0 (0) | 0 (0) |
|  | No number of participants reported | 136 (45) | 1 (2) | 56 (47) | 38 (51) | 15 (45) | 26 (90) |
| Review outcomes listed in Methods section | At least one outcome stated | 234 (78) | 45 (100) | 99 (83) | 49 (66) | 28 (85) | 13 (45) |
|  | Median (IQR) number of outcomes | 4 (2-6) | 6 (5-9) | 4 (2-6) | 1 (1-3) | 2 (2-4) | 4 (2-5) |
| Primary outcome specified | Explicitly stated | 112 (37) | 43 (96) | 53 (45) | 9 (12) | 6 (18) | 1 (3) |
|  | Only one outcome reported | 24 (8) | 0 (0) | 3 (3) | 21 (28) | 0 (0) | 0 (0) |
|  | NA (diagnostic test accuracy SR) | 12 (4) | 0 (0) | 0 (0) | 0 (0) | 12 (36) | 0 (0) |
|  | Not stated | 152 (51) | 2 (4) | 63 (53) | 44 (59) | 15 (45) | 28 (97) |
| Type of primary outcome | Dichotomous | 91/136 (67) | 27/43 (63) | 36/56 (64) | 25/30 (83) | 3/6 (50) | 0 (0) |
|  | Continuous | 29/136 (21) | 10/43 (23) | 16/56 (29) | 2/30 (7) | 0 (0) | 1/1 (100) |
|  | Rate | 5/136 (4) | 2/43 (5) | 1/56 (2) | 2/30 (7) | 0 (0) | 0 (0) |
|  | Time-to-event | 9/136 (7) | 4/43 (9) | 2/56 (4) | 0 (0) | 3/6 (50) | 0 (0) |
|  | Other (e.g. prevalence, not specified) | 2/136 (1) | 0 (0) | 1/56 (2) | 1/30 (3) | 0 (0) | 0 (0) |
| Statistical significance of primary outcome intervention effect | Favourable, statistically significant | 53/136 (39) | 18/43 (42) | 35/56 (63) | 0 (0) | 0 (0) | 0 (0) |
|  | Favourable, statistically non-significant | 23/136 (17) | 12/43 (28) | 11/56 (20) | 0 (0) | 0 (0) | 0 (0) |
|  | Unfavourable, statistically significant | 0 (0) | 0 (0) | 0 (0) | 0 (0) | 0 (0) | 0 (0) |
|  | Unfavourable, statistically non-significant | 9/136 (7) | 6/43 (14) | 3/56 (5) | 0 (0) | 0 (0) | 0 (0) |
|  | Direction of effect unclear | 4/136 (3) | 0 (0) | 3/56 (5) | 0 (0) | 0 (0) | 1/1 (100) |
|  | No comparison of two interventions | 38/136 (28) | 0 (0) | 2/56 (4) | 30/30 (100) | 6/6 (100) | 0 (0) |
|  | Outcome not measured in included studies, or P-values not calculated | 9/136 (7) | 7/43 (16) | 2/56 (4) | 0 (0) | 0 (0) | 0 (0) |
| Meta-analysis performed | Yes | 189 (63) | 32 (71) | 78 (66) | 49 (66) | 25 (76) | 5 (17) |
|  | No | 104 (35) | 8 (18) | 40 (34) | 25 (34) | 7 (21) | 24 (83) |
|  | NA (< 2 included studies) | 7 (2) | 5 (11) | 1 (1) | 0 (0) | 1 (3) | 0 (0) |
| Meta-analysis model used | Fixed-effect model for all meta-analyses | 34/189 (18) | 14/32 (44) | 12/78 (15) | 4/49 (8) | 2/25 (8) | 2/5 (40) |
|  | Random-effects model for all meta-analyses | 89/189 (47) | 7/32 (22) | 40/78 (51) | 25/49 (51) | 15/25 (60) | 2/5 (40) |
|  | Varied | 54/189 (29) | 11/32 (34) | 21/78 (27) | 16/49 (33) | 6/25 (24) | 0 (0) |
|  | Not reported | 12/188 (6) | 0 (0) | 5/78 (6) | 4/49 (8) | 2/25 (8) | 1/5 (20) |
| Study risk of bias/quality assessment incorporated into meta-analysis | Yes (e.g. via subgroup or sensitivity analysis) | 31/189 (16) | 4/32 (13) | 11/78 (14) | 10/49 (20) | 4/25 (16) | 2/5 (40) |
|  | No | 112/189 (59) | 28/32 (87) | 52/78 (67) | 20/49 (41) | 11/25 (44) | 1/5 (40) |
|  | NA - no risk of bias (or quality) assessment | 46/189 (24) | 0 (0) | 15/78 (19) | 19/49 (39) | 10/25 (40) | 2/5 (40) |
| Statistical heterogeneity investigated | Using statistical methods or qualitatively assessed (e.g. via narrative discussion) | 207/300 (69) | 33/45 (73) | 86/119 (72) | 52/74 (70) | 28/33 (85) | 8/29 (28) |
|  | Using statistical methods when meta-analysis performed | 175/189 (93) | 32/32 (100) | 71/78 (91) | 45/49 (92) | 24/25 (96) | 3/5 (60) |
| Methods to evaluative statistical heterogeneity | Visual inspection of the forest plot | 17/189 (9) | 9/32 (28) | 4/78 (5) | 2/49 (4) | 1/25 (4) | 1/5 (20) |
|  | Chi-square or Cochran's Q | 119/189 (63) | 19/32 (59) | 47/78 (60) | 32/49 (65) | 20/25 (80) | 1/5 (20) |
|  | I^2^ | 161/189 (85) | 30/32 (94) | 67/78 (86) | 40/49 (82) | 21/25 (84) | 3/5 (60) |
|  | tau^2^ | 12/189 (6) | 3/32 (9) | 7/78 (9) | 0 (0) | 1/25 (4) | 1/5 (20) |
|  | Other (Galbraith plot or Fisher exact test) | 3/189 (2) | 0 (0) | 0 (0) | 2/49 (4) | 1/25 (4) | 0 (0) |
| Heterogeneity statistic guided choice of meta-analysis model | Yes (e.g. random-effects model selected if I^2^ >50%) | 72/189 (38) | 8/32 (25) | 27/78 (35) | 22/49 (45) | 15/25 (60) | 0 (0) |
| Risk of publication bias assessed | Formally assessed (e.g. funnel plot, sensitivity analysis) | 93 (31) | 7 (16) | 39 (33) | 33 (45) | 13 (39) | 1 (3) |
|  | Not assessed, but authors planned to if they identified a sufficient number of studies | 37 (12) | 28 (62) | 7 (6) | 0 (0) | 2 (6) | 0 (0) |
|  | No plan to assess publication bias | 170 (57) | 10 (22) | 73 (61) | 41 (55) | 18 (55) | 28 (97) |
| Appropriateness of statistical methods used to infer publication bias | Method used in meta-analysis with sufficient number of studies (n≥10) | 53 (18) | 6 (13) | 21 (18) | 19 (26) | 6 (18) | 1 (3) |
|  | Method used in meta-analysis with insufficient number of studies (n<10) | 40 (13) | 1 (2) | 18 (15) | 14 (19) | 7 (21) | 0 (0) |
|  | Method not used, and would have been inappropriate to do so (e.g. number of studies less than 10) | 169 (56) | 33 (73) | 67 (56) | 33 (45) | 12 (36) | 24 (83) |
| Methods used to infer publication bias (if meta-analysis performed) | Funnel plot | 84/93 (90) | 6/7 (86) | 37/39 (95) | 29/33 (88) | 11/13 (85) | 1/1 (100) |
|  | Egger's test | 52/93 (56) | 0 (0) | 15/39 (38) | 26/33 (79) | 10/13 (77) | 1/1 (100) |
|  | Begg's test | 34/93 (37) | 0 (0) | 10/39 (26) | 20/33 (61) | 4/13 (31) | 0 (0) |
|  | Trim and fill method | 6/93 (6) | 0 (0) | 4/39 (10) | 0 (0) | 1/13 (8) | 1/1 (100) |
|  | Fail-safe method | 2/93 (2) | 0 (0) | 1/39 (3) | 0 (0) | 1/13 (8) | 0 (0) |
|  | Other (Macaskill test, sensitivity analysis) | 4/93 (4) | 1/7 (14) | 0 (0) | 2/33 (6) | 1/13 (8) | 0 (0) |
| Appropriateness of funnel plot | Appropriate (meta-analysis includes at least 10 studies) | 48/84 (57) | 5/6 (83) | 19/37 (51) | 17/29 (59) | 6/11 (55) | 1/1 (100) |
|  | Inappropriate (meta-analysis includes less than 10 studies) | 36/84 (43) | 1/6 (17) | 18/37 (49) | 12/29 (41) | 5/11 (45) | 0 (0) |
| Risk of publication bias discussed/considered | Narratively in results, discussion or conclusion section | 141 (47) | 29 (64) | 55 (46) | 37 (50) | 18 (55) | 2 (7) |
| Additional analyses | Subgroup analysis (if meta-analysis performed) | 87/189 (46) | 12/32 (38) | 40/78 (51) | 23/49 (47) | 8/25 (32) | 4/5 (80) |
|  | Sensitivity analysis (if meta-analysis performed) | 92/189 (49) | 12/32 (38) | 43/78 (55) | 27/49 (55) | 9/25 (36) | 1/5 (20) |
|  | Meta-regression (if meta-analysis performed) | 21/189 (11) | 0 (0) | 7/78 (9) | 10/49 (20) | 2/25 (8) | 2/5 (40) |
|  | Network meta-analysis | 7 (2) | 0 (0) | 7 (6) | 0 (0) | 0 (0) | 0 (0) |
|  | Individual participant data meta-analysis | 2 (1) | 2 (4) | 0 (0) | 0 (0) | 0 (0) | 0 (0) |
|  | Other (cumulative meta-analysis, thematic synthesis, multivariate meta-analysis, trial sequential analysis, unweighted pooling) | 11 (4) | 1 (2) | 3 (3) | 5 (7) | 1 (3) | 1 (3) |
|  | No additional analyses | 162 (54) | 29 (64) | 58 (49) | 30 (41) | 20 (61) | 25 (86) |
| Harms analysed | Data on harms/adverse events reported | 106/164 (65) | 37 (82) | 69 (58) | NA | NA | NA |
|  | Planned to but none of the included studies measured harms/adverse events | 7/164 (4) | 4 (9) | 3 (3) | NA | NA | NA |
|  | No plan to analyse harms/adverse events | 51/164 (31) | 4 (9) | 47 (40) | NA | NA | NA |
| Cost-effectiveness data analysed | Cost-effectiveness data reported | 16/172 (9) | 2 (4) | 6 (5) | NA | NA | 8/8 (100) |
|  | Planned to but none of the included studies measured cost-effectiveness | 7/172 (4) | 5 (11) | 2 (2) | NA | NA | 0 (0) |
|  | No plan to analyse cost-effectiveness | 149/172 (87) | 38 (84) | 111 (93) | NA | NA | 0 (0) |
| GRADE assessment reported | GRADE assessment in a Summary of Findings table | 29 (10) | 26 (58) | 3 (3) | 0 (0) | 0 (0) | 0 (0) |
|  | GRADE assessment in text only | 3 (1) | 1 (2) | 1 (1) | 0 (0) | 1 (3) | 0 (0) |
|  | Planned to but no eligible studies | 1 (1) | 1 (2) | 0 (0) | 0 (0) | 0 (0) | 0 (0) |
|  | No plan to perform GRADE assessment | 267 (89) | 17 (38) | 115 (97) | 74 (100) | 32 (97) | 29 (100) |
| Limitations reported | Both limitations at study level and review level were reported | 173 (58) | 32 (71) | 63 (53) | 43 (58) | 23 (70) | 12 (41) |
|  | Only limitations at study level reported | 67 (22) | 10 (22) | 31 (26) | 15 (20) | 5 (15) | 6 (21) |
|  | Only limitations at review level reported | 27 (9) | 0 (0) | 10 (8) | 8 (11) | 3 (9) | 6 (21) |
|  | No limitations reported | 33 (11) | 3 (7) | 15 (13) | 8 (11) | 2 (6) | 5 (17) |
| Limitations incorporated into therapeutic SR conclusions | Study risk of bias/quality/limitations incorporated into abstract conclusion | 99/164 (60) | 42/45 (93) | 57/119 (48) | NA | NA | NA |
|  | Study risk of bias/quality/limitations not incorporated into abstract conclusion | 65/164 (40) | 3/45 (7) | 62/119 (52) | NA | NA | NA |
| Conflicts of interest of review authors reported | Yes | 260 (87) | 45 (100) | 103 (87) | 61 (82) | 30 (91) | 21 (72) |
|  | No | 40 (13) | 0 (0) | 16 (13) | 13 (18) | 3 (9) | 8 (28) |
| Conflicts of interest or funding of authors of included studies reported | Yes | 21/296 (7) | 13/42 (31) | 7/118 (6) | 0 (0) | 0 (0) | 1 (3) |
|  | No | 275/296 (93) | 29/42 (69) | 111/118 (94) | 74 (100) | 33 (100) | 28 (97) |
| Source of funding of the SR | Non-profit | 142 (47) | 38 (84) | 48 (40) | 30 (41) | 9 (27) | 17 (59) |
|  | For-profit | 8 (3) | 0 | 3 (3) | 2 (3) | 0 (0) | 3 (10) |
|  | Mixed | 2 (1) | 0 | 1 (1) | 1 (1) | 0 (0) | 0 (0) |
|  | Authors specified there was no funding | 39 (13) | 5 (11) | 14 (12) | 11 (15) | 7 (21) | 2 (7) |
|  | Not reported | 109 (36) | 2 (4) | 53 (45) | 30 (41) | 17 (51) | 7 (24) |

^a^Denominator of fractions indicates the number of reports where the variable concerned was considered relevant to the SR. Illustrative binomial 95% confidence intervals for percentages when sample size is 300: 1% (0.2% to 3%); 5% (3% to 8%); 10% (7% to 14%); 25% (20% to 30%); 50% (44% to 56%); 75% (70% to 80%)
